# Synergistic Interactions between HDAC and Sirtuin Inhibitors in Human Leukemia Cells

**DOI:** 10.1371/journal.pone.0022739

**Published:** 2011-07-27

**Authors:** Michele Cea, Debora Soncini, Floriana Fruscione, Lizzia Raffaghello, Anna Garuti, Laura Emionite, Eva Moran, Mirko Magnone, Gabriele Zoppoli, Daniele Reverberi, Irene Caffa, Annalisa Salis, Antonia Cagnetta, Micaela Bergamaschi, Salvatore Casciaro, Ivana Pierri, Gianluca Damonte, Filippo Ansaldi, Marco Gobbi, Vito Pistoia, Alberto Ballestrero, Franco Patrone, Santina Bruzzone, Alessio Nencioni

**Affiliations:** 1 Department of Internal Medicine, University of Genoa, Genoa, Italy; 2 Jerome Lipper Multiple Myeloma Center, Dana-Farber Cancer Institute, Harvard Medical School, Boston Novartis Institutes for BioMedical Research, Cambridge, Massachusetts, United States of America; 3 Advanced Biotechnology Center (ABC), Genoa, Italy; 4 Laboratory of Oncology, IRCCS G. Gaslini, Genoa, Italy; 5 Animal Facility, National Cancer Institute, Genoa, Italy; 6 Section of Biochemistry, Department of Experimental Medicine, University of Genoa, Genoa, Italy; 7 Medical Oncology Division C, National Cancer Institute, Genoa, Italy; 8 Department of Hematology and Oncology, San Martino Hospital, Genoa, Italy; 9 Department of Health Sciences, University of Genoa, Genoa, Italy; Chinese University of Hong Kong, Hong Kong

## Abstract

Aberrant histone deacetylase (HDAC) activity is frequent in human leukemias. However, while classical, NAD^+^-independent HDACs are an established therapeutic target, the relevance of NAD^+^-dependent HDACs (sirtuins) in leukemia treatment remains unclear. Here, we assessed the antileukemic activity of sirtuin inhibitors and of the NAD^+^-lowering drug FK866, alone and in combination with traditional HDAC inhibitors. Primary leukemia cells, leukemia cell lines, healthy leukocytes and hematopoietic progenitors were treated with sirtuin inhibitors (sirtinol, cambinol, EX527) and with FK866, with or without addition of the HDAC inhibitors valproic acid, sodium butyrate, and vorinostat. Cell death was quantified by propidium iodide cell staining and subsequent flow-cytometry. Apoptosis induction was monitored by cell staining with FITC-Annexin-V/propidium iodide or with TMRE followed by flow-cytometric analysis, and by measuring caspase3/7 activity. Intracellular Bax was detected by flow-cytometry and western blotting. Cellular NAD^+^ levels were measured by enzymatic cycling assays. Bax was overexpressed by retroviral transduction. Bax and SIRT1 were silenced by RNA-interference. Sirtuin inhibitors and FK866 synergistically enhanced HDAC inhibitor activity in leukemia cells, but not in healthy leukocytes and hematopoietic progenitors. In leukemia cells, HDAC inhibitors were found to induce upregulation of Bax, a pro-apoptotic Bcl2 family-member whose translocation to mitochondria is normally prevented by SIRT1. As a result, leukemia cells become sensitized to sirtuin inhibitor-induced apoptosis. In conclusion, NAD^+^-independent HDACs and sirtuins cooperate in leukemia cells to avoid apoptosis. Combining sirtuin with HDAC inhibitors results in synergistic antileukemic activity that could be therapeutically exploited.

## Introduction

Histone deacetylases (HDACs) regulate the acetylation status of histones and other intracellular substrates. Four classes of HDACs have been identified, three of which are NAD^+^-independent HDACs (class I, II, and IV, referred to here as classical HDACs; and their inhibitors as HDAC inhibitors) [1], [2]. The recently discovered class III HDACs are sirtuins (SIRT1-7) [3]. Mammalian sirtuins are homologs of the yeast silent information regulator 2 (Sir2), and are characterized by a unique NAD^+^-dependent enzymatic activity [4].

Classical HDACs have long been known for their involvement in cancer, including leukemias [1], [2]. Aberrant HDAC activity is commonly observed in leukemia cells, leading to skewed gene expression, increased proliferation, and resistance to apoptosis [1], [2]. HDAC inhibitors, some of which have been available for decades, show antileukemic activity *in vitro* and in animal models, and thus underwent clinical evaluations, mostly for acute myelogenous leukemia (AML) and myelodysplastic syndromes [5], [6], [7], [8], [9]. Overall, these agents are very well tolerated, which makes them particularly suited for treating elderly patients or patients with relevant co-morbidities. However, although the most recent inhibitors, such as vorinostat and romidepsin, appear to be more active than traditional valproic acid (VA), HDAC inhibitors alone will rarely induce disease remissions, their benefit being mostly limited to hematological improvements [5], [6], [7], [8], [9]. Thus, strategies to increase their efficacy are warranted.

Recently, sirtuins, particularly SIRT1, have also been proposed to play a role in leukemogenesis [10]. SIRT1 was found to be overexpressed in AML and in B-cell chronic lymphocytic leukemia (B-CLL), and downregulated during neutrophil differentiation of acute promyelocytic leukemia cells [11], [12], [13]. It was reported that SIRT1 antagonizes PML-induced cellular senescense [14]. Moreover, increased SIRT1 levels were detected in chemoresistant leukemia cells and in imatinib-resistant chronic myelogenous leukemia cells [10], [15]. The mechanisms invoked to explain SIRT1's oncogenic activity are mostly related to its role in cell defenses and survival in response to stress. SIRT1 directly deacetylates, and consequently inactivates, p53 [16], [17]. Moreover, SIRT1 prevents apoptosis in response to damage or stress by interfering with the activity of the FOXO family of transcription factors, of Bax, Rb, and of E2F1 [10].

Sirtuins are virtually unaffected by all HDAC inhibitors currently available [18]. However, numerous small-molecule sirtuin inhibitors have been described, several of which show anticancer activity in preclinical models [10], [19]. Moreover, nicotinamide phosphoribosyltransferase (Nampt) inhibitors, such as FK866 [20], by lowering intracellular NAD^+^ concentrations, deprive sirtuins of their substrate and thus reduce their activity [21]. Indeed, in many instances, pharmacological Nampt inhibition has been shown to recreate the biological consequences of sirtuin obstruction or genetic deletion [20], [21], [22], [23], [24], [25].

In this study, we evaluated sirtuin inhibitors and FK866, either alone or in combination with HDAC inhibitors, for their antileukemic activity. To this end, we made use of a large cohort of: primary leukemia cells; leukemia cell lines; healthy leukocytes and hematopoietic progenitors. Our results indicate that sirtuins and classical HDACs cooperate in leukemia cells to prevent apoptosis. Combined inhibition of the two types of HDACs results in a synergistic antileukemic activity with potential to have clinical applications.

## Results

### Sirtuin inhibitors synergistically enhance HDAC inhibitor activity in human leukemia cells

**Figure 1:**
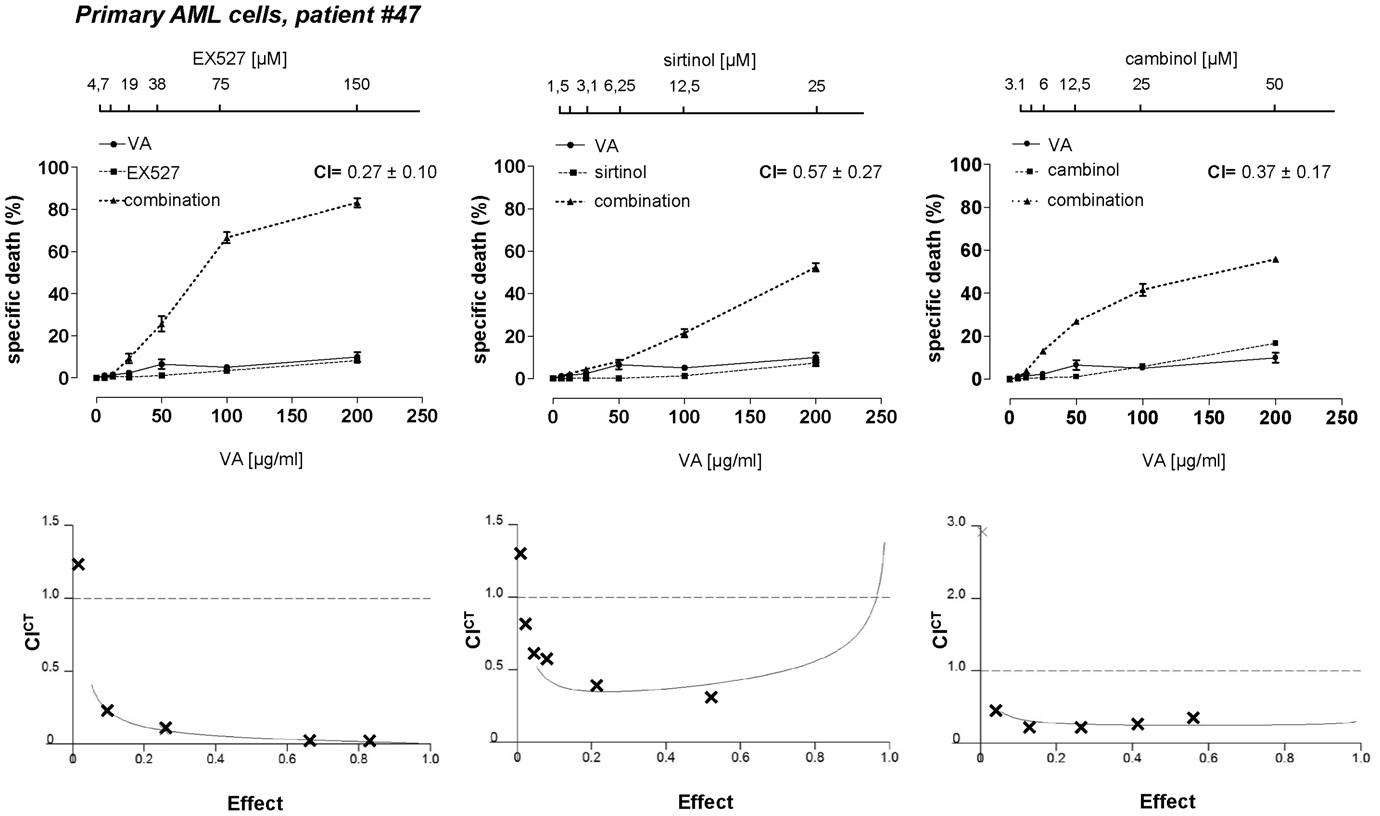
HDAC and sirtuin inhibitors synergistically kill primary AML cells. Primary AML cells were incubated with or without sirtuin inhibitors (EX527, sirtinol, cambinol) in the presence or absence of VA at the indicated concentrations. Viability was assessed 48 h later by PI cell staining and flow cytometry. CI values refer to the highest drug concentrations used. CI^CT^s for each drug combination are presented in the lower insets. An effect of 1 corresponds to 100% specific death whereas an effect of 0 corresponds to 0% specific death.

We investigated the antileukemic activity of the sirtuin inhibitors sirtinol, cambinol, and EX527. Sirtinol and cambinol are reported to inhibit SIRT1 and SIRT2 [10]. EX527 selectively inhibits SIRT1 when used at concentration in the nanomolar or low-micromolar range, whilst at higher drug concentrations it also inhibits SIRT2 (IC_50_, 19.6 µM) and SIRT3 (IC_50_, 48.7 µM) [26]. Sirtuin inhibitors were either used alone or in combination with the HDAC inhibitors VA and butyrate (BU). These inhibitors were tested on a large cohort of primary AML and B-CLL samples. In addition, for additional titration and follow-up experiments we made use of the leukemia cells lines U937 (AML), 697 (pre-B-cell leukemia), and Jurkat (acute T-cell leukemia). Finally, healthy peripheral blood mononuclear cells (PBMCs) were also treated with these drug combinations. Cell viability was assessed after a 48 h treatment by standard propidium iodide (PI) staining and flow cytometry. Throughout these experiments, sirtuin inhibitors and HDAC inhibitors were found to have partial cytotoxic activity in leukemia cells when used as single agents (with specific cell deaths that were typically <50%) (Figure 1, S1, S2, S3, and Tables S1, S2, S3, S4). Co-administration of an HDAC inhibitor with a sirtuin inhibitor resulted in a synergistic enhancement of their cytotoxic activity, as shown by calculation of both cooperative index (CI) and combination index according to Chou and Talalay statistics (CI^CT^) [27], [28]. On the contrary, in healthy PBMCs, these drugs were not only poorly active, but they also failed to show any cooperation (Figure S4). These data indicate that inhibition of SIRT1 (and possibly of other sirtuins affected by the concentrations of inhibitors tested) has *per se* limited cytotoxic activity in leukemia cells. However, sirtuin inhibitors and HDAC inhibitors potentiate each other's activity.

To confirm the role of SIRT1 inhibition in the synergy between sirtuin and HDAC inhibitors in leukemia cells we silenced this sirtuin member in Jurkat cells by transfecting the cells in the presence of a SIRT1-specific siRNA or a non-targeting siRNA as a control (Figure S5A). Indeed, SIRT1 silencing increased HDAC inhibitor-induced cell death (Figure S5B).

Finally, we sought to determine whether SIRT1 expression would predict the efficacy of the combination sirtuin inhibitor/HDAC inhibitor. To this end, we determined SIRT1 levels by quantitative PCR (QPCR) in the primary leukemia samples and in the leukemia cell lines used and compared these to SIRT1 expression in healthy PBMCs. Although with some variability among samples, SIRT1 expression in primary leukemia cells (B-CLL and AML) was found to be similar to that observed in healthy leukocytes (Figure S6A). Conversely, in U937, Jurkat, and 697 cells, SIRT1 was expressed at lower levels as compared to PBMCs (Figure S6B). Finally, in B-CLL cells, which represented the largest available group of samples, no correlation between cytotoxic activity or CI of the combination sirtuin inhibitor plus HDAC inhibitor or Nampt inhibitor plus HDAC inhibitor (see below) was observed (Figure S7, S8). Thus, SIRT1 levels as detected by QPCR do not appear to be predictive of the activity of combined sirtuin and HDAC inhibition.

### Combined HDAC and sirtuin inhibitors trigger the apoptotic cascade in leukemia cells

**Figure 2:**
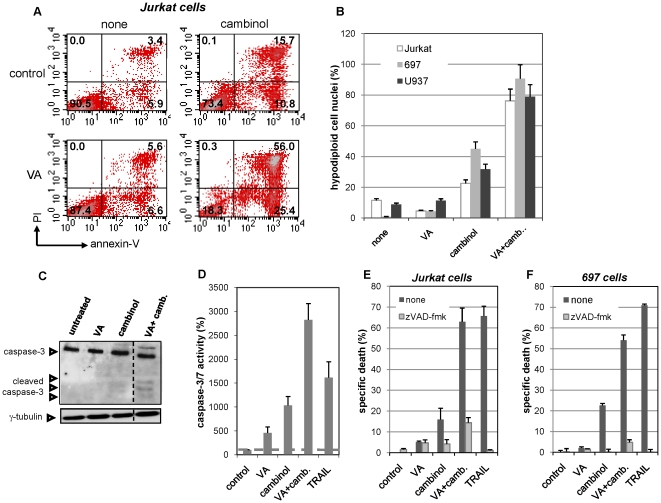
Combined HDAC and sirtuin inhibition synergistically induces apoptosis in leukemia cells. A–D 1×10^6^ Jurkat, 697, or U937 cells/well were plated in 6-well plates and treated for 48 h with our without 50 µM cambinol, 100 µg/ml VA, or their combination. Thereafter, cells were harvested, washed, and used for Annexin-V/PI staining and flow cytometry (A), flow cytometric quantification of hypodiploid cell nuclei (B), protein lysates preparation for caspase-3 and γ-tubulin detection by immunoblotting (C, 697 cells), detection of caspase-3/7 enzymatic activity (D, 697 cells). E, F, Jurkat cells and 697 cells were plated in 96-well plates and pre-incubated for 1 h with 100 µM zVAD-fmk. Thereafter, 50 µM cambinol, 100 µg/ml VA, their combination, or 100 ng/ml TRAIL were added to the medium. Viability was assessed 48 h later by flow cytometry. A, C, one representative experiment out of three is shown. B, D–F, results are means ± SD of three separate experiments.

Cambinol alone and, to a higher degree, in combination with VA, produced changes in Jurkat, 697 and U937 cells that are typically observed during apoptosis, including: an increase in the percentage of Annexin-V^+^/PI^−^ (early apoptotic) and Annexin-V^+^/PI^+^ (late apoptotic) cells; the appearance of hypodiploid cell nuclei; caspase-3 cleavage and a consistent increase in caspase-3/-7 enzymatic activity (Figure 2A–D and data not shown). To formally assess the role of caspase activity in leukemia cell death in response to the stimuli under investigation, we made use of the pan-caspase inhibitor zVAD-fmk. Since the latter strongly reduced cell death in response to sirtuin inhibitors and to their combination with HDAC inhibitors (Figure 2E, F, Figure S9), we concluded that these compounds kill leukemia cells via caspase-mediated apoptosis.

### HDAC inhibitor-induced Bax upregulation is involved in leukemia cells sensitization to sirtuin inhibitors

**Figure 3:**
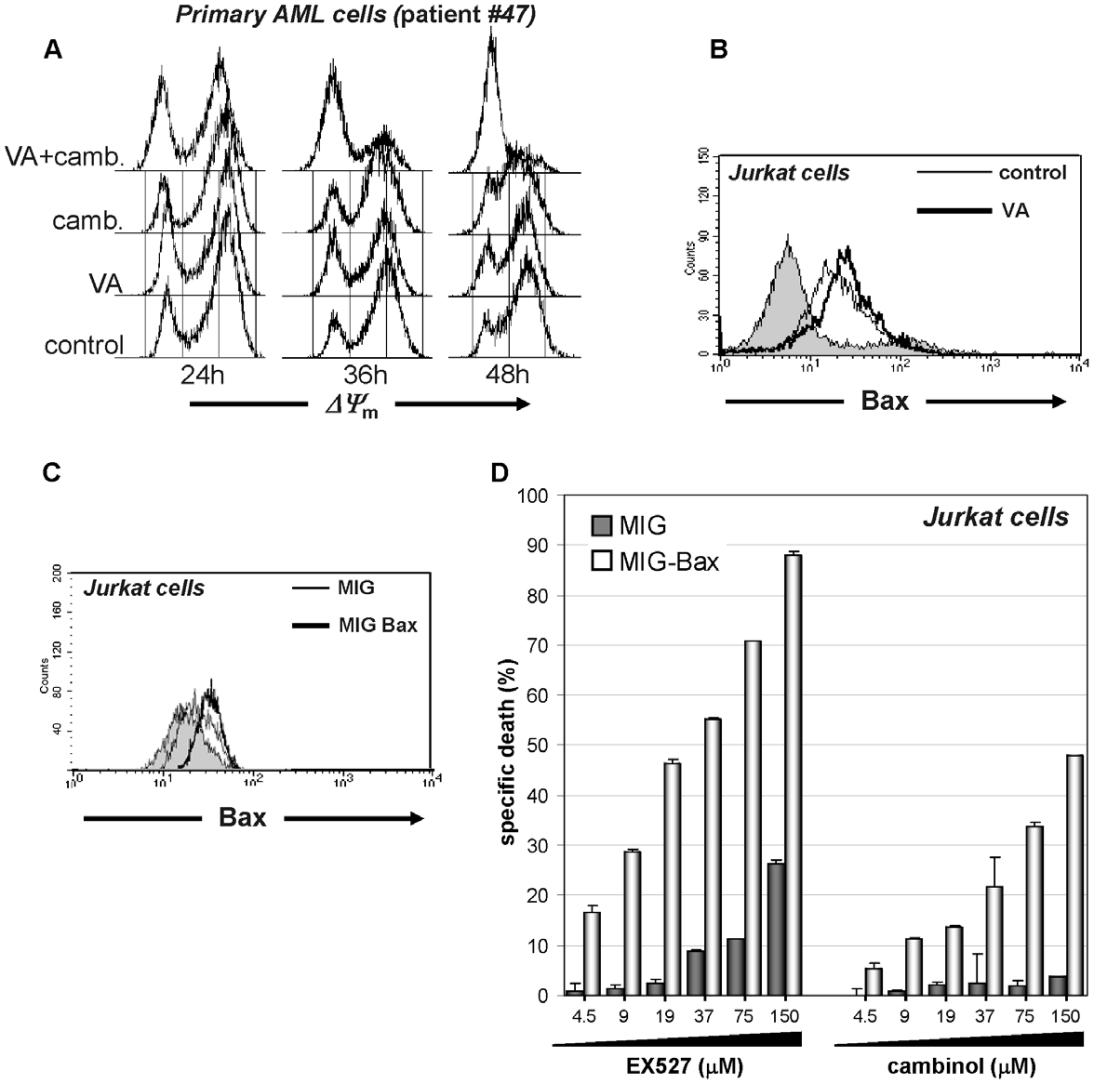
HDAC inhibitor-induced Bax upregulation contributes to the synergy with sirtuin inhibitors. A, 3×10^6^ primary AML cells/well were plated in 6-well plates and incubated in the presence or absence of 50 µM cambinol, 100 µg/ml VA, or their combination. *ΔΨ*_m_ was monitored at the indicated time points by TMRE staining and flow cytometry. B, 1×10^6^ Jurkat cells were plated in 6-well plates and incubated for 48 h in the presence or absence of 100 µg/ml VA. Thereafter, intracellular Bax content was determined by flow cytometry. C, D, Jurkat cells were transduced with either pMIG or pMIG-Bax. Infected cells were FACS sorted, allowed to expand, and subsequently used for flow cytometric detection of intracellular Bax (C) and for viability assays (D). For these, pMIG- or pMIG-Bax-transduced Jurkat were plated in 96-well plates and incubated in the presence or absence of EX527 or cambinol at the indicated concentrations. Viability was determined by PI staining and flow cytometry 48 h later. A–C, one representative experiment out of three is presented. D, Results are means ± SD of three separate experiments.

**Figure 4:**
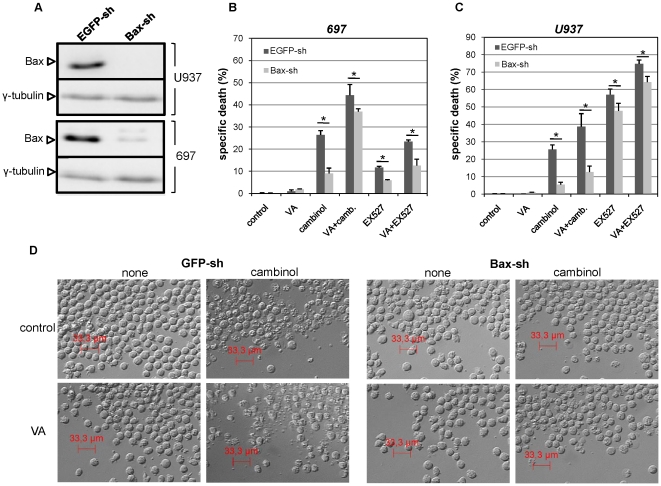
Bax silencing reduces sirtuin inhibitor activity in leukemia cells. A, B, U937 and 697 cells were transduced to either express an anti-EGFP shRNA (EGFP-sh) or a validated anti-Bax shRNA (Bax-sh). Thereafter cells were used for cell lysate preparation and Bax and γ-tubulin were detected by immunoblotting. B, EGFP-sh or Bax-sh 697 cells were incubated with or without 100 µg/ml VA, 75 µM EX527, 50 µM cambinol, or their combinations. Viability was assessed 36 h later by PI staining and flow cytometry. C, D, EGFP-sh or Bax-sh U937 cells were incubated with or without 100 µg/ml VA, 150 µM EX527, 100 µM cambinol, or their combinations. 36 h later, cells were imaged by light microscopy (D) and cell death was determined by flow cytometric quantification of PI-positive cells (C). A, D, one representative experiment out of three is presented. B, C, Results are means ± SD of three separate experiments. *: p<0.05.

Apoptotic cell death can be initiated by different mechanisms [29]. Irreversible damage of intracellular components usually results in activation of the intrinsic mitochondrial apoptotic pathway. Conversely, the surface death receptor pathway is normally initiated by extracellular stimuli, although autocrine activation mechanisms have also been proposed for this apoptotic route. Using tetramethylrhodamine ethyl ester (TMRE) cell staining, we found that cambinol induced mitochondrial transmembrane potential (*ΨΔ*_m_) dissipation in leukemia cells, and that VA strongly enhanced this effect, suggesting that the mitochondrial apoptotic machinery is activated in response to these stimuli (Figure 3A and data not shown). To gain insight into this phenomenon, we focused on the pro-apoptotic Bcl-2 family member Bax, since this protein plays a key role in mitochondrial permeability transition pore formation and is also an established target of SIRT1's anti-apoptotic activity [10], [30], [31]. Namely, SIRT1 induces Bax sequestration away from mitochondria by promoting its interaction with Ku70 [30], [31]. Moreover, Bax expression is known to be down-regulated by HDACs and, accordingly, HDAC inhibitors induce Bax upregulation [18], [32], [33], [34]. Indeed, using flow cytometry and western blotting we found increased Bax levels in VA-treated Jurkat cells (Figure 3B, S10A). Similarly, VA increased Bax amounts in U937 and 697 cells (Figure S10A). Conversely, in healthy PBMCs, VA failed to induce Bax upregulation (Figure S10B). Since previous experiments indicated that SIRT1 inhibition induces apoptosis in the presence of Bax overexpression, we hypothesized that Bax accumulation mediated by HDAC inhibitors, compounded by sirtuin inhibition, could be a vital factor making leukemia cells especially susceptible to mitochondrial damage and subsequent apoptosis observed in response to these drugs. To confirm that increased Bax levels would enhance cell death via SIRT1 inhibition, we retrovirally engineered Jurkat cells to overexpress Bax (Figure 3C). Indeed, Jurkat cells with increased Bax levels were highly predisposed to cell demise upon treatment with the sirtuin inhibitors EX527 and cambinol (Figure 3D). Finally, to formally define Bax's role in the cytotoxic activity of sirtuin inhibitors and of their combination with HDAC inhibitors, we silenced Bax in 697 and in U937 cells with a validated anti-Bax shRNA (Figure 4A). Cells engineered to express an anti-EGFP shRNA were used as a control. As predicted, in cells with reduced Bax levels, cell death in response to sirtuin inhibitors alone or in combination with VA was reduced (Figure 4B–D), thus confirming the role of this pro-apoptotic protein in cell death in response to these stimuli.

### NAD^+^-biosynthesis inhibition with FK866 synergistically enhances HDAC inhibitor activity in leukemia cells

Sirtuins depend on NAD^+^ for their enzymatic activity [3], [4], [21]. The Nampt inhibitor FK866 impairs sirtuin activity by reducing intracellular NAD^+^ availability, as shown by the observation that SIRT1 targets are hyperacetylated in FK866-treated cells [35]. Since FK866 (as well as other Nampt inhibitors) has already undergone preclinical and clinical studies [36], [37], we aimed to assess whether the same level of synergy observed with combined sirtuin and HDAC inhibitors would be seen when replacing the sirtuin inhibitors with FK866.

**Figure 5:**
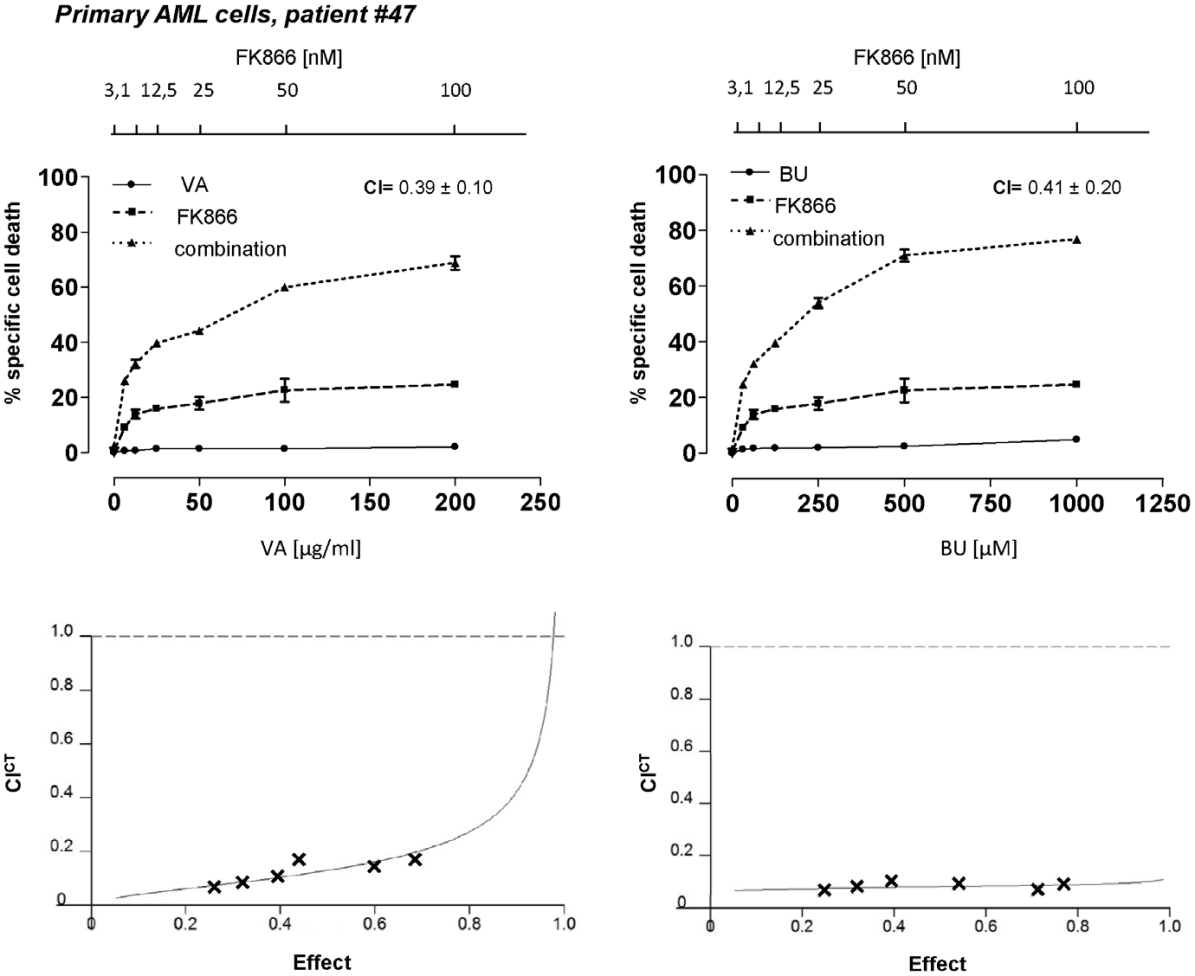
FK866 and HDAC inhibitors synergistically kill primary B-CLL cells. Primary B-CLL cells were incubated with or without FK866 at the indicated concentrations for 48 h. Thereafter, VA or vorinostat were added at the indicated concentrations. Viability was assessed 48 h later by PI cell staining and flow cytometry. CI values refer to the highest drug concentrations used. CI^CT^s for each drug combination are presented in the lower insets.

**Figure 6:**
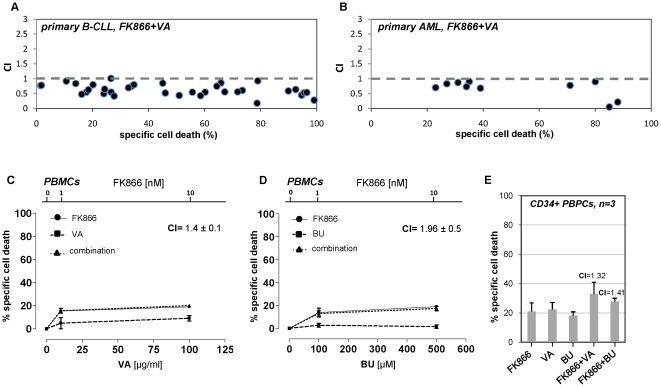
FK866/HDAC inhibitor synergistic interaction spares healthy leukocytes and CD34^+^ PBPCs. A, B, Primary B-CLL or AML cells were incubated with or without 10 nM FK866 for 48 h. Thereafter, 100 µg/ml VA was added (or not) to the medium. 48 h later, dead cells were quantified by PI-staining and flow-cytometry. CIs for the combination FK866/VA were calculated and plotted vs. the specific cell death induced by the same drug combination in each sample. C, D, Healthy PBMCs were incubated with or without FK866 at the indicated concentrations for 48 h. Thereafter, VA or BU were added at the indicated concentrations. Viability was assessed 48 h later by PI cell staining and flow cytometry. CI values refer to the highest drug concentrations used. E, Healthy CD34^+^ PBPCs were incubated in 96-well plates and treated with 10 nM FK866 for 48 h. Subsequently, 100 µg/ml VA or 500 µM BU were added where indicated. Viability was assessed 48 h later. CI values for the drug combinations are indicated inside the graph area. C–E, Results are means of three separate experiments with three different donors.

Treatment with FK866 effectively reduced intracellular NAD^+^ concentration in leukemia cells, whereas the HDAC inhibitor VA failed to diminish intracellular NAD^+^ content (Figure S11A). Moreover, as shown in Figure S11B, FK866-induced cell death was reversed by supplementation with exogenous NAD^+^, thus confirming that the mode of action of this drug is related to NAD^+^-depletion. In primary AML cells, primary B-CLL cells, and in the leukemia cell lines, FK866 enhanced the cytotoxic activity of the HDAC inhibitors in a synergistic manner (Figure 5, 6A–B, S12, S13, S14, S15, and Table S5). In Figure 6A, B, the CIs of the combination FK866/VA in primary leukemia samples (AML and B-CLL) are plotted vs. the specific cell deaths caused by this drug combination. The raw viability data of these measurements as well as the results obtained with the combination FK866/BU are presented in Table S5. Noticeably, we found that the broad spectrum HDAC inhibitor vorinostat also synergistically interacted with FK866 in primary leukemia cells and in leukemia cell lines (Figure S12, S13, S14, and data not shown) [1], thus confirming the findings obtained with VA and BU. Finally, in healthy PBMCs and in CD34^+^ peripheral blood precursor cells (PBPCs), the synergistic interaction between FK866 and the HDAC inhibitors (VA or BU) was not observed (Figure 6C–E). Therefore, these results are consistent with FK866 recreating the antileukemic activity of sirtuin inhibitors and their capacity to potentiate HDAC inhibitor-induced cell death in leukemia cells.

## Discussion

Our data indicate that sirtuin and HDAC inhibitors cooperate to the killing of human leukemia cells. A two-pronged mechanism is shown to contribute to this form of synergy. On the one hand, HDAC inhibitors upregulate the pro-apoptotic Bcl2-family protein Bax. In turn, this condition predisposes leukemia cells to apoptotic cell demise when SIRT1 is inhibited. These findings are in line with previous studies which showed that SIRT1 prevents Bax-mediated apoptosis by causing its cytoplasmic sequestration by Ku70, and that SIRT1 blockade results in initiation of the intrinsic apoptotic pathway in the presence of Bax overexpression [30], [31].

We confirmed Bax's role in the synergy between sirtuin and HDAC inhibitors in leukemia cells by overexpressing it and by showing that increased Bax amounts indeed augment the efficacy of sirtuin inhibitors. Moreover, silencing Bax by stable RNA interference was found to reduce the activity of sirtuin inhibitors and of their combination with VA. However, it was of interest to observe that, despite efficient Bax silencing, the activity of sirtuin inhibitors, alone or coupled to VA, was not fully abolished. These findings suggest that Bax-independent mechanisms may also play a role in the antileukemic activity of these drugs. This is not surprising given how broadly sirtuin- and HDAC-mediated protein modifications are predicted to affect protein expression and activity, resulting in increased predisposition to apoptotic programs in malignant cells [1], [2], [10].

The Nampt inhibitor FK866 reduces SIRT1 activity by diminishing intracellular NAD^+^ levels [21]. Studies show that FK866 has antileukemic activity *in vitro* and in leukemia and lymphoma animal models [38], [39]. Our experiments indicate that indeed FK866 behaves similarly to sirtuin inhibitors in terms of cytotoxic activity and cooperation with HDAC inhibitors in leukemia cells. Therefore, since Nampt inhibitors for clinical uses are already available and have shown to be well tolerated [36], [37], these could in principle replace sirtuin inhibitors in combination protocols with HDAC inhibitors. Importantly, since the concentrations of FK866, VA, BU, and vorinostat used in our experiments are within the pharmacological range [8], [36], [40], [41], these drug combinations are predicted to also show activity in patients.

Audrito and colleagues have recently reported that SIRT1 inhibition with nicotinamide has cytotoxic activity on B-CLL cells, and that this effect requires the presence of wild type p53 [13]. Previous studies showed that SIRT1 deacetylates p53, thereby preventing its transcriptional activity [16], [17]. Thus, SIRT1 inhibition was proposed to upregulate several p53-dependent pro-apoptotic factors in B-CLL cells, thereby promoting apoptosis. In our case, functional p53 did not appear to be required for the synergy between sirtuin inhibitors (or FK866) and HDAC inhibitors, since this form of cooperation was also observed in primary B-CLL cells with 17p deletion. Moreover, Jurkat cells, which carry a mutant p53, were also highly susceptible to the combination of sirtuin and HDAC inhibitors. Nonetheless, it remains conceivable that, at least in some of the cases we studied, increased p53-mediated transcription via SIRT1 inhibition did contribute to the observed synergistic cytotoxicity.

It has to be noticed that, although we confirmed SIRT1's role in the synergy between sirtuin and HDAC inhibitors by RNAi-mediated SIRT1 silencing, we cannot in principle exclude that inhibition of other sirtuin members could also play a role in this synergy. As a matter of fact, the sirtuin inhibitors used in this study are not specific for SIRT1 and can also inhibit other sirtuins, including SIRT2, SIRT3, and, possibly, SIRT6 [10], [24], [25], [26]. The same applies to the Nampt inhibitor FK866 [20], [21], [22], [23], [24], [25]. SIRT6 involvement in the synergy with HDAC inhibitors is unlikely since Jurkat cells where SIRT6 had been silenced by RNA-interference failed to exhibit increased susceptibility to HDAC inhibitors (data not shown). We suggest that the potential of other sirtuins as targets for treating leukemias is further investigated.

Combined sirtuin and HDAC inhibitors (or FK866 and HDAC inhibitors) showed antileukemic activity against cells of different lineages (primary AML, primary B-CLL, U937, 697, and Jurkat cells), suggesting that such drug combinations may find applications in a broad spectrum of hematological malignancies. Interestingly, as opposite to what was observed in leukemia cells, HDAC and sirtuin inhibitors were poorly active and failed to show any cooperation in CD34^+^ hematopoietic progenitors and in PBMCs. For classical HDAC inhibitors, preferential activity against malignant tissues has been reported [2], [18]. The fact that cancer cells frequently express higher amounts of certain HDACs, and a peculiar composition of the HDAC complexes in malignant cells have both been proposed as possible reasons for this selectivity [2], [18]. In contrast to Audrito and co-workers [13], we failed to detect increased SIRT1 expression in B-CLL cells as compared to healthy leukocytes. This could be due to the fact that these authors compared B-CLL cells to healthy B cells, while in our case SIRT1 expression in B-CLL cells was compared to its levels in PBMCs (which are mostly CD3^+^ T cells). However, as a possible explanation for the preferential activity of combined sirtuin and HDAC inhibitors in leukemias, we found that HDAC inhibition increases Bax's levels in leukemia cells, but not in healthy leukocytes. Thus, it is likely that, by removing one arm of the two-pronged mechanism that we found underlie this form of synergy, the cooperation between the two types of agents is disabled. Further studies should address the specificity of sirtuin and HDAC inhibitors for leukemic cells. However, regardless of the underlying mechanism, these data highlight a specific requirement for sustained sirtuin and HDAC activity by leukemia cells and suggest a possible Achilles' heel of leukemias that could be exploited therapeutically.

In conclusion, sirtuin inhibitors (or FK866) and HDAC inhibitors cooperate in turning off cellular mechanisms that protect leukemia cells from apoptosis. Co-administration of sirtuin and HDAC inhibitors should be further examined for clinical applications.

## Materials and Methods

### Ethics statement

The collection of peripheral blood samples from leukemia patients and healthy volunteers for *in vitro* assays and drug testing was approved by the Ethics Committee of the San Martino Hospital (Genoa, Italy). Samples were collected following written informed consent obtainment according to the Declaration of Helsinki.

### Cell lines and reagents

U937, Jurkat, 293T, and Phoenix cells were obtained from ATCC (LGC Standards, Teddington, UK). 697 cells were purchased from German Collection of Microorganisms and Cell Cultures (DSMZ, Braunschweig, Germany). Cells were grown in RPMI 1640-based medium supplemented with 10% FBS and antibiotics. Sirtinol, cambinol, VA, BU, NAD^+^, and TMRE were purchased from Sigma-Aldrich (Sigma Aldrich Italia, Milano, Italy). EX527 was from Tocris Bioscience (Bristol, UK). Vorinostat was from Selleck Chemicals LLC. zVAD-fmk was from Merck Chemicals (Nottingham, UK). FK866 was generously provided by the NIMH Chemical Synthesis and Drug Supply Program.

### Primary cell isolation

Peripheral blood samples were obtained from healthy donors (n = 10) and patients with B-CLL (n = 36) or AML (n = 12) at the Department of Internal Medicine of the University of Genoa, Genoa, Italy. The clinical and laboratory features of the B-CLL and AML patients are summarized in Table S6 and S7, respectively. B-CLL cells were isolated by density gradient centrifugation with Ficoll-Hypaque (Biotest, Dreieich, Germany). The B-CLL phenotype of the obtained cell preparations was confirmed by immunostaining with anti-CD19, anti-CD5, and anti-CD23 (Immunotech, Marseille, France), and subsequent flow cytometric analysis. The purity of the isolated B-CLL cells was typically >85%. AML blasts were isolated by adding a 6% dextran solution (Fresenius Kabi, Varese, Italy) to the blood specimens at a ratio of 4∶5, followed by 1-h incubation at room temperature. Thereafter, the leukocytes-enriched supernatants were transferred to a 50 ml conical centrifuge tube and centrifuged at 300× g for 10 min. Residual red blood cells were lysed by resuspending the cell pellets in 4 ml 0.2% NaCl followed by addition of 4 ml 1.6% NaCl and immediate centrifugation at 300× g for 10 min. Normal PBMCs were isolated from healthy donor blood samples by density gradient centrifugation with Ficoll-Hypaque. Cells were either used immediately for viability assays, or stored at −80° in medium containing 20% FBS and 10% DMSO. CD34^+^ peripheral blood precursor cells (PBPCs) were obtained from excess PBPC concentrates (1–2 ml) from G-CSF-mobilized patients undergoing autologous PBPC transplantation, following informed consent according to the Declaration of Helsinki. CD34^+^ cells were purified using the CD34 MicroBead Kit from Miltenyi Biotec (Bergisch Gladbach, Germany) following the manufacturer's instructions. Using this method, CD34^+^ cells were typically >80% pure and >80% viable as detected by PI staining and flow cytometry.

### Viability assays

2×10^5^ cells/well (primary B-CLL and AML cells, PBMCs, PBPCs) or 5×10^4^ cells/well (U937, 697, Jurkat cells) were plated in 96 well plates in a final volume of 200 µl in the presence or absence of the indicated stimuli. Dead cells were quantified at the indicated times by PI staining (2 µg/ml) and flow cytometry (FACS Calibur, Becton Dickinson, BD Italia, Milan, Italy). Specific death was calculated as follows: [(% experimental death−% spontaneous death)/(100−% spontaneous death)]×100. FITC-Annexin-V-PI staining was done with the Annexin-V-FITC Kit from BioVision (Mountain View, CA, USA) according to the manufacturer's instructions. For the detection of hypodiploid cell nuclei, cell pellets were resuspended in a buffer containing 0.1% sodium citrate, 0.1% Triton-X 100, and 50 µg/ml PI. Thereafter, cells were analyzed by flow cytometry.

### ΔΨ_m_ determination

Cells were harvested, washed, and incubated in the presence of 50 nM TMRE in regular RPMI-based medium for 15 min at 37°C. Thereafter, cells were analyzed by flow cytometry.

### Immunoblotting

Cell lysates were generated from 1.5×10^6^ cells by directly resuspending cell pellets in sodium dodecyl sulfate (SDS) sample buffer (Tris-HCl 0.25 M, pH 6.8, SDS 2%, glycerol 10%, β-mercaptoethanol 2%, bromophenol blue 0.005%; Boston Bioproducts, Boston, MA). Cell lysates were immediately boiled at 100°C for 10 minutes and stored at −20°C for subsequent use. Proteins were separated on an SDS-polyacrylamide gel and electroblotted to a polyvinylidene difluoride (PVDF) membrane (Pall Gelman Laboratory, Ann Arbor, MI). Proteins were visualized by probing the membranes with the following antibodies: anti-caspase-3, anti-SIRT1 (Cell Signaling Technology), anti-Bax, (rabbit polyclonal, Santa Cruz Biotechnology), and anti-γ tubulin (mouse monoclonal, Sigma Aldrich).

### Caspase activity assays

Caspase activity was determined with the Apo-ONE Caspase-3/7 Kit from Promega according to the manufacturer's instructions.

### Immunostaining and flow cytometric detection of intracellular Bax

For intracellular detection of Bax levels, cells were fixed in 2% paraformaldehyde for 10 min at room temperature. Cells were subsequently washed and permeabilized using 0.5% Triton X-100 in PBS for 5 min at room temperature. Thereafter, cells were washed and incubated with rabbit anti-human Bax polyclonal antibody (BD Biosciences, USA) for 30 min at 4°C according to the manufacturer's instructions. Thereafter, cells were washed and incubated with a FITC-conjugated rabbit anti-mouse antibody (a kind gift of Dr. Alessandro Poggi, National Cancer Institute, Genoa, Italy) for 30 min at 4°C. Finally, cells were analyzed by flow cytometry.

### Retroviral transduction

Phoenix cells were plated in 4 ml medium in 6 cm-dishes and allowed to adhere for 24 h. Thereafter, cells were transfected with 4 µg plasmid DNA (pMIG, Dr. William Hahn, Dana Farber Cancer Institute, Boston, MA, USA; or pMIG-Bax, Dr. Stanley J. Korsmeyer, Dana Farber Cancer Institute, Boston, MA, US, both from Addgene, Cambridge MA, USA) using Transit 293 (Mirus Bio, Madison, WI, USA). The viral supernatant was harvested 36 and 48 h later and used to infect Jurkat cells in 24-well plates in the presence of 5 µg/ml protamine sulfate. pMIG- and pMIG-Bax-infected Jurkat cells were FACS-sorted for EGFP expression using a FACSAria Cell Sorter (Becton Dickinson, BD Italia).

For lentivirus-mediated shRNA delivery, 293T cells were used as a packaging cell line. 7×10^5^ cells were plated in 4 ml medium in 6 cm-dishes, allowed to adhere for 24 h, and subsequently transfected with 2 µg MISSION EGFP shRNA Control Vector (Sigma Aldrich; pLKO.1 variant; short hairpin sequence 5′-CCGGTACAACAGCCACAACGTCTATCTCGAGATGGTTCTGATCAGTTCCGGCTTTTTG-3′) or MISSION Bax shRNA Vector (Sigma Aldrich; pLKO.1 variant; short hairpin sequence 5′-CCGGGCCGGAACTGATCAGAACCATCTCGAGATGGTTCTGATCAGTTCCGGCTTTTTG-3′), 0.7 µg VSVG-, 0.7 µg gag/pol-, and 0.7 µg env-encoding plasmids using Transit 293. The viral supernatant was harvested 36 and 72 h later and used to infect U937 and 697 cells in 24-well plates in the presence of 5 µg/ml protamine sulfate. Infected cells were selected using 1 µg/ml puromycin.

### siRNA transfection

Jurkat cells were transfected using the Nucleofector System (Amaxa GmbH, Cologne, Germany), with StealthTM duplex short interference RNAs (siRNA) targeting SIRT1, or scrambled siRNA (StealthTM Negative Control), all from Dharmacon (Lafayette, CO).

### Determination of intracellular NAD^+^ levels

To determine intracellular NAD^+^ content, 2×10^6^ cells, treated or not, were harvested and lysed with 0.6 M perchloric acid (PCA). Acid extracts were neutralized and the intracellular content of NAD^+^ was assessed with a sensitive enzymatic cycling assay [42]. NAD^+^ value was normalized to protein concentration (Bradford assay, Bio-Rad Laboratories).

### Q-PCR

Total RNA was extracted from 5×10^5^ cells using RNeasy kit reagents (Qiagen, Qiagen Italia, Milan, Italy). Total RNA (1 µg) was reverse transcribed using random hexamers in a final volume of 50 µl. 5 µl of the resulting cDNA was used for Q-PCR using a TaqMan 7900 HT Fast Real TimeAB. Pre-designed primers and probes for SIRT1, and RPLP0 were obtained from Applied Biosystems. Gene expression was normalized to housekeeping gene expression (RPLP0). Comparisons in gene expression were done using the 2^−ΔΔCt^ method.

### Light Microscopy

Cells were imaged at room temperature using with the 20× magnification of a Zeiss AXIOVERT200 microscope, an Olympus C-4040ZOOM camera, and the software Olympus CAMEDIA Master 2.5.

### Statistical analysis

Each experiment was performed at least three times. The cooperative index (CI) was calculated as the sum of the specific cell deaths induced by the single agents divided by the specific cell death in response to the combination. CI values <1,  = 1 and >1 indicate a synergistic, additive or infra-additive effect respectively [27]. The Combination Index according to Chou and Talalay (CI^CT^) was calculated with Calcusyn Version 2.1 software (Biosoft, Cambridge, UK): CI^CT^ = C_1,x_/IC_x,1_+C_2,x_/IC_x,2_. C_1,x_ and C_2,x_ are the concentrations of the first and of the second compound, respectively, which achieve x% drug effect when the compounds are used in combination [28]. IC_x,1_ and IC_x,2_ are the single agent concentrations required for the same effect. A CI^CT^<1 is considered indicative of a synergistic drug interaction. GraphPad Prism version 4.00 (GraphPad Software, San Diego,CA) was used for the *t*-tests and to calculate the Pearson correlation coefficients.

## Supporting Information

Figure S1 **EX527 and VA synergistically kill primary B-CLL cells.** Primary B-CLL cells were plated in 96-well plates and incubated with or without EX527 and VA at the indicated concentrations. Viability was assessed 48 h later by PI cell staining and flow cytometry. The CI value refers to the highest drug concentrations used.
(PDF)

Figure S2 **EX527 and VA cooperate in 697 cells.** 697 cells were incubated with or without EX527 or VA at the indicated concentrations. Viability was assessed 48 h later by PI cell staining and flow cytometry. CI values refer to the highest drug concentrations used. The CI^CT^s for the different drug combinations are presented in the lower insets.
(PDF)

Figure S3 **Sirtuin inhibitors and HDAC inhibitors synergistically kill Jurkat cells.** A–D, Jurkat cells were incubated in 96-well plates with or without EX527, cambinol, BU, or VA at the indicated concentrations. Viability was assessed 48 h later by PI cell staining and flow cytometry. CI values refer to the highest drug concentrations used.
(PDF)

Figure S4 **Sirtuin inhibitors and HDAC inhibitors show poor activity and fail to cooperate in healthy PBMCs.** A–C, Healthy PMBCs were incubated in 96-well plates with or without sirtinol, cambinol, BU, or VA at the indicated concentrations. Viability was assessed 48 h later by PI cell staining and flow cytometry. CI values refer to the highest drug concentrations used. Results are means ± SD of three independent experiments with three different donors.
(PDF)

Figure S5 **SIRT1 silencing enhances HDAC inhibitor activity in Jurkat cells.** A, B. Jurkat cells were transfected with a non-targeting siRNA (cntr siRNA) or with an anti-SIRT1-siRNA. Thereafter, two days later, cells were used for protein lysate preparation or plated in 96-well plates for viability assays. A, SIRT1 and γ-tubulin levels were determined by immunoblotting. B, Cells were incubated in the presence or absence of the indicated concentrations of VA or of BU. Two days later, dead cells were quantified by PI staining and flow cytometry. One representative experiment out of three is presented. *: p<0.05.
(PDF)

Figure S6 **SIRT1 expression in primary leukemia cells and in leukemia cell lines.** A, RNA was extracted from freshly isolated normal PBMCs (n = 10), primary B-CLL cells (n = 36), AML cells (n = 11), and from the cell lines U937, Jurkat, and 697. SIRT1 levels were determined by Q-PCR. SIRT1 levels in primary leukemia samples (A) and in cell lines (B) were compared to the mean value obtained in PBMCs using the 2^−ΔΔCT^ method.
(PDF)

Figure S7 **Correlation between SIRT1 expression and activity of the combination sirtuin/HDAC inhibitors in leukemia cells.** Primary B-CLL cells were plated in 96 well plates and treated with or without 100 µg/ml VA, 500 µM BU, 50 µM cambinol, or their combinations. Dead cells were enumerated two days later by PI staining and flow cytometry. The correlation between the CI (A, B) or the overall cytotoxic activity (C, D) of each drug combination and SIRT1 expression was assessed by Pearson correlation coefficient.
(PDF)

Figure S8 **Correlation between SIRT1 expression and activity of the combination FK866/HDAC inhibitors in leukemia cells.** Primary B-CLL cells were treated with or without 10 nM FK866, 100 µg/ml VA, 500 µM BU, or their combination. Dead cells were enumerated four days later by PI staining and flow cytometry. The correlation between the CI (A, B) or the overall cytotoxic activity (C, D) of each drug combination and SIRT1 expression was assessed by Pearson correlation coefficient.
(PDF)

Figure S9 **zVAD-fmk reduces cell death in response to sirtuin and HDAC inhibitors in leukemia cells.** A, Jurkat cells were pre-incubated for 1 h with or without 100 µM zVAD-fmk. Thereafter, 100 µg/ml VA, 30 µM sirtinol, 75 µM EX527, or their combinations were added as indicated. Viability was assessed two days later by PI staining and flow cytometry. B–D, 697 cells were pre-incubated for 1 h with or without 100 µM zVAD-fmk. Thereafter, 100 µg/ml VA, 30 µM sirtinol, or their combination were added as indicated. Two days later, cells were imaged by light microscopy (D), dead cells were enumerated by PI staining and flow cytometry (B), and hypodiploid cell nuclei were counted by PI staining of isolated cell nuclei and flow cytometry (C).
(PDF)

Figure S10 **VA induces Bax upregulation in leukemia cell lines, but not in healthy PBMCs.** A, 3×10^6^ Jurkat, U937, and 697 cells were plated in 6-well plates in the presence or absence of 100 µg/ml VA. Two days later, cells were harvested, washed and used for protein lysate preparation. Bax and γ-tubulin levels were determined by immunoblotting. B, 10^7^ PBMCs were plates in 3 ml medium in 6-well plates in the presence or absence of the indicated concentrations of VA. Two days later, cells were harvested, washed, and used for cell lysate preparation. Bax and γ-tubulin expression were determined by immunoblotting. A, B one representative experiment out of three is shown.
(PDF)

Figure S11 **FK866-mediated NAD^+^ depletion mediates FK866's cytotoxic activity.** A, Primary B-CLL and AML cells were incubated in 24-well plates in the presence or absence of 10 nM FK866, 100 µg/ml VA, 500 µM BU, or their combination. 48 h later, NAD^+^ levels were determined by enzymatic cycling assay. NAD^+^ values were normalized to protein content (expressed in mg). B, Primary AML cells were plated in 96-well plates and incubated with 100 nM FK866. NAD^+^ was added to the medium every 12 h in order to achieve the indicated concentrations. Viability was quantified after 96 h of incubation by PI staining and flow-cytometry.
(PDF)

Figure S12 **FK866 and HDAC inhibitors synergistically kill primary B-CLL cells.** Primary B-CLL cells were incubated with or without FK866 at the indicated concentrations for 48 h. Thereafter, VA or vorinostat were added at the indicated concentrations. Viability was assessed 48 h later by PI cell staining and flow cytometry. CI values refer to the highest drug concentrations used. CI^CT^s for each drug combination are presented in the lower insets.
(PDF)

Figure S13 **Synergistic interaction between FK866 and HDAC inhibitors in the AML cell line U937.** U937 cells were incubated with or without FK866 at the indicated concentrations for 48 h. Thereafter, VA, vorinostat, or BU were added at the indicated concentrations. Viability was assessed 48 h later by PI cell staining and flow cytometry. CI values refer to the highest drug concentrations used. CI^CT^s for each drug combination are shown in the lower insets.
(PDF)

Figure S14 **Synergistic interaction between FK866 and HDAC inhibitors in Jurkat cells.** Jurkat cells were incubated with or without FK866 at the indicated concentrations for 48 h. Thereafter, VA, vorinostat, or BU were added at the indicated concentrations. Viability was assessed 48 h later by PI cell staining and flow cytometry. CI values refer to the highest drug concentrations used. CI^CT^s for each drug combination are presented in the lower insets.
(PDF)

Figure S15 **Synergistic interaction between FK866 and VA in 697 pre-B-cell leukemia cells.** 697 cells were incubated with or without FK866 at the indicated concentrations for 48 h. Thereafter, VA was added at the indicated concentrations. Viability was assessed 48 h later by PI cell staining and flow cytometry. CI values refer to the highest drug concentrations used. CI^CT^s are shown in the lower inset.
(PDF)

Table S1 **Synergistic interactions between cambinol and VA in primary leukemia cells.** Primary B-CLL (#3, #9, #11, #12, #13, #19, #24, #27, #33) or AML (#41, #42, #46) cells were plated in 96 well plates and stimulated with 100 µg/ml VA, cambinol (camb.) at the indicated concentrations, or their combinations. Specific cell death was detected four days later by flow cytometry. CIs are indicated in parenthesis. ND: not determined.
(PDF)

Table S2 **Synergistic interactions between cambinol and BU in primary leukemia cells.** Primary B-CLL cells were plated in 96 well plates and stimulated with 500 µM BU, cambinol (camb.) at the indicated concentrations, or their combinations. Specific cell death was detected four days later by flow cytometry. CIs are indicated in parenthesis.
(PDF)

Table S3 **Synergistic interactions between sirtinol and VA in primary leukemia cells.** Primary B-CLL (#3, #9, #11, #12, #13, #19, #24, #27, #33, #36) or AML (#41, #42, #46) cells were plated in 96 well plates and stimulated with 100 µg/ml VA, sirtinol (sirt.) at the indicated concentrations, or their combinations. Specific cell death was detected four days later by flow cytometry. CIs are indicated in parenthesis.
(PDF)

Table S4 **Synergistic interactions between sirtinol and BU in primary leukemia cells.** Primary B-CLL (#3, #9, #11, #12, #13, #19, #24, #27, #33) or AML (#41, #42) cells were plated in 96 well plates and stimulated with 500 µM BU, sirtinol (sirt.) at the indicated concentrations, or their combinations. Specific cell death was detected four days later by flow cytometry. CIs are indicated in parenthesis.
(PDF)

Table S5 **Synergistic interactions between FK866 and HDAC inhibitors in primary leukemia cells.** Primary B-CLL (#1–35) or AML (#37–45) cells were plated in 96 well plates and stimulated with 100 µg/ml VA, 500 µM BU, 10 nM FK866 or their combinations. Specific cell death was detected four days later by PI staining and flow cytometry. CIs are indicated in parenthesis. ND: not determined.
(PDF)

Table S6 **Clinical and laboratory features of patients with B-CLL.** ND: not determined; Nor, normal; +: trisomy; *:chemonaive patient.
(PDF)

Table S7 **Clinical and laboratory features of patients with AML.** PB: peripheral blood.
(PDF)
