# Supplementary material for: Synergistic Interactions between HDAC and Sirtuin Inhibitors in Human Leukemia Cells
Source: PLoS One. 2011 Jul 27;6(7):e22739. doi: 10.1371/journal.pone.0022739 (PMC3144930; doi:10.1371/journal.pone.0022739)
Supplement: Figure S1 — EX527 and VA synergistically kill primary B-CLL cells. Primary B-CLL cells were plated in 96-well plates and incubated with or without EX527 and VA at the indicated concentrations. Viability was assessed 48 h later by PI cell staining and flow cytometry. The CI value refers to the highest drug concentrations used. (PDF) [file pone.0022739.s001.pdf]

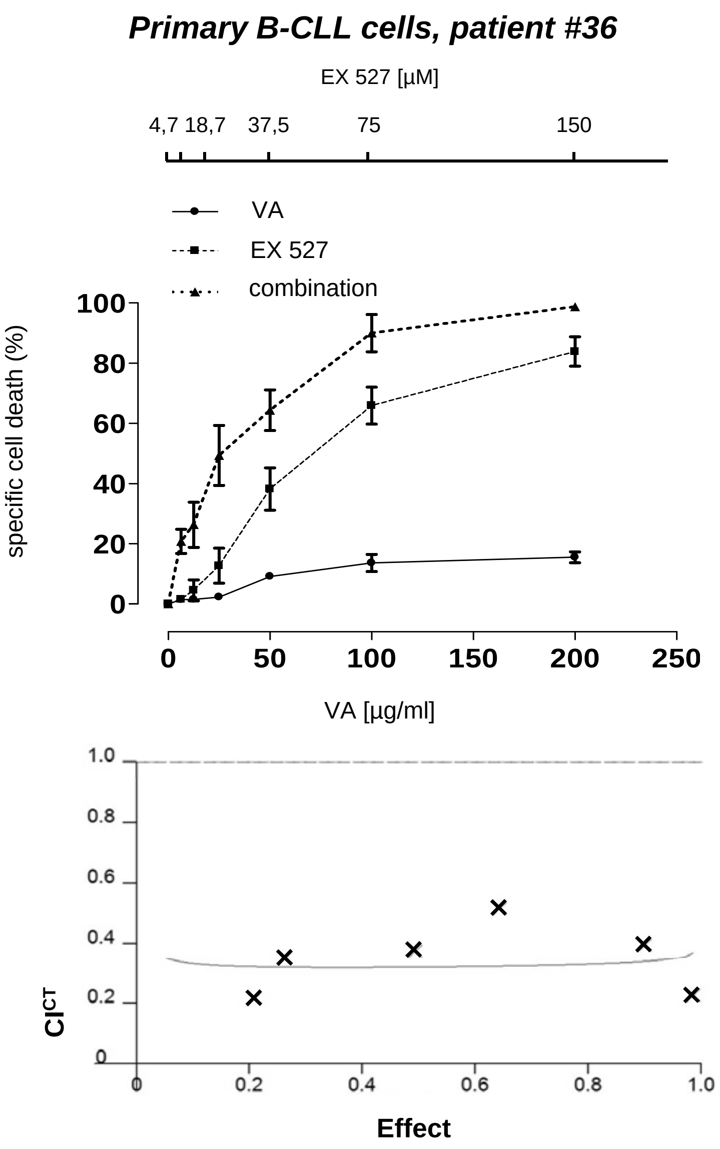

Figure S1. EX527 and VA synergistically kill primary B-CLL cells. Primary B-CLL cells were plated in 96-well plates and incubated with or without EX527 and VA at the indicated concentrations. Viability was assessed 48 h later by PI cell staining and flow cytometry. The CI value refers to the highest drug concentrations used.
