# Supplementary material for: Synergistic Interactions between HDAC and Sirtuin Inhibitors in Human Leukemia Cells
Source: PLoS One. 2011 Jul 27;6(7):e22739. doi: 10.1371/journal.pone.0022739 (PMC3144930; doi:10.1371/journal.pone.0022739)
Supplement: Figure S2 — EX527 and VA cooperate in 697 cells. 697 cells were incubated with or without EX527 or VA at the indicated concentrations. Viability was assessed 48 h later by PI cell staining and flow cytometry. CI values refer to the highest drug concentrations used. The CICTs for the different drug combinations are presented in the lower insets. (PDF) [file pone.0022739.s002.pdf]

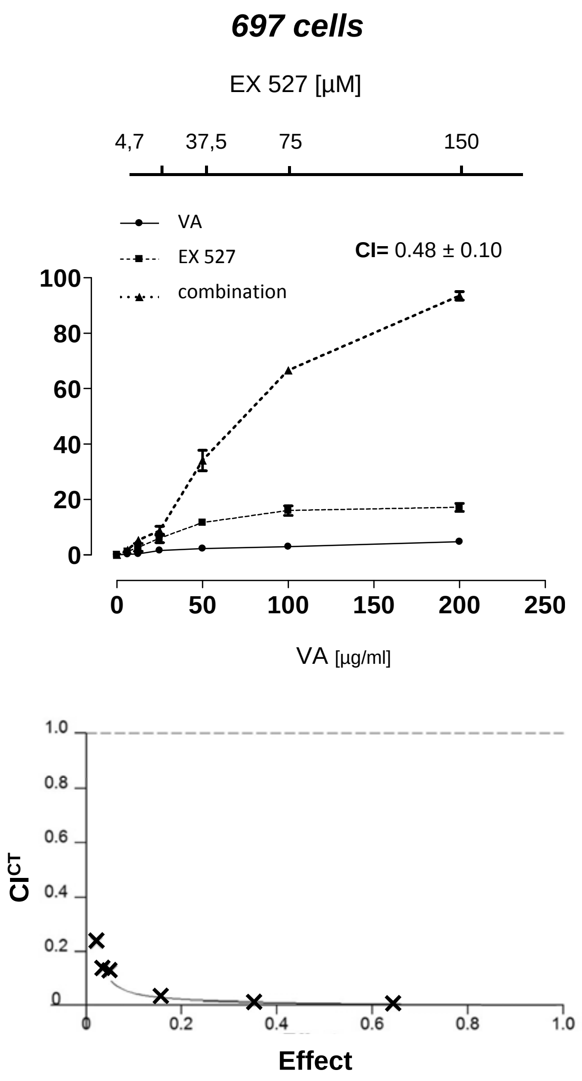


**Figure S2. EX527 and VA cooperate in 697 cells.** 697 cells were incubated with or without EX527 or VA at the indicated concentrations. Viability was assessed 48 h later by PI cell staining and flow cytometry. CI values refer to the highest drug concentrations used. The $CI^{CT}$s for the different drug combinations are presented in the lower insets.
