# Supplementary material for: Synergistic Interactions between HDAC and Sirtuin Inhibitors in Human Leukemia Cells
Source: PLoS One. 2011 Jul 27;6(7):e22739. doi: 10.1371/journal.pone.0022739 (PMC3144930; doi:10.1371/journal.pone.0022739)
Supplement: Figure S3 — Sirtuin inhibitors and HDAC inhibitors synergistically kill Jurkat cells. A–D, Jurkat cells were incubated in 96-well plates with or without EX527, cambinol, BU, or VA at the indicated concentrations. Viability was assessed 48 h later by PI cell staining and flow cytometry. CI values refer to the highest drug concentrations used. (PDF) [file pone.0022739.s003.pdf]

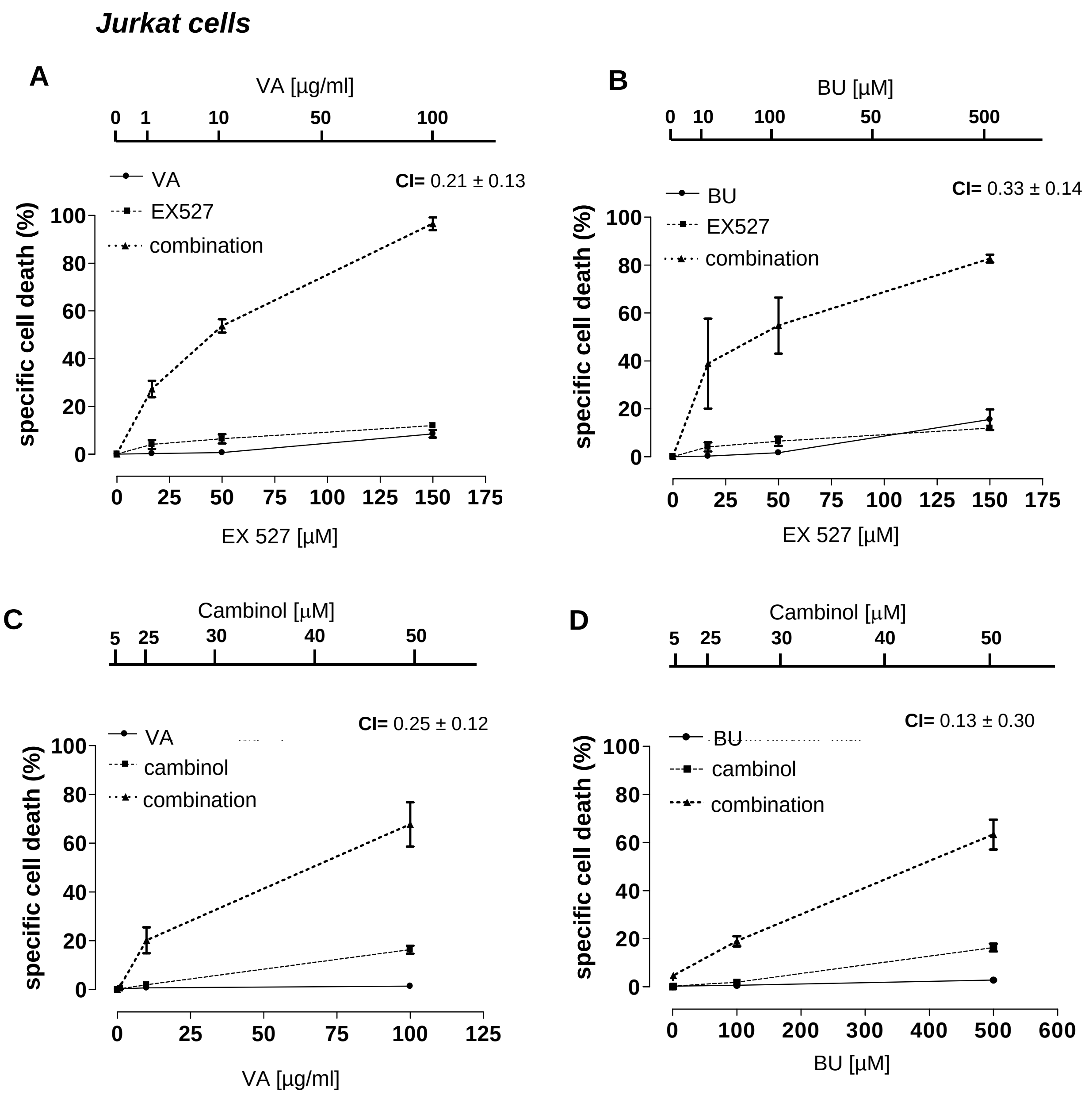

Figure S3. Sirtuin inhibitors and HDAC inhibitors synergistically kill Jurkat cells. A-D, Jurkat cells were incubated in 96-well plates with or without EX527, cambinol, BU, or VA at the indicated concentrations. Viability was assessed 48 h later by PI cell staining and flow cytometry. CI values refer to the highest drug concentrations used.
