# Supplementary material for: Synergistic Interactions between HDAC and Sirtuin Inhibitors in Human Leukemia Cells
Source: PLoS One. 2011 Jul 27;6(7):e22739. doi: 10.1371/journal.pone.0022739 (PMC3144930; doi:10.1371/journal.pone.0022739)
Supplement: Figure S4 — Sirtuin inhibitors and HDAC inhibitors show poor activity and fail to cooperate in healthy PBMCs. A–C, Healthy PMBCs were incubated in 96-well plates with or without sirtinol, cambinol, BU, or VA at the indicated concentrations. Viability was assessed 48 h later by PI cell staining and flow cytometry. CI values refer to the highest drug concentrations used. Results are means ± SD of three independent experiments with three different donors. (PDF) [file pone.0022739.s004.pdf]

healthy PBMCs, n=3

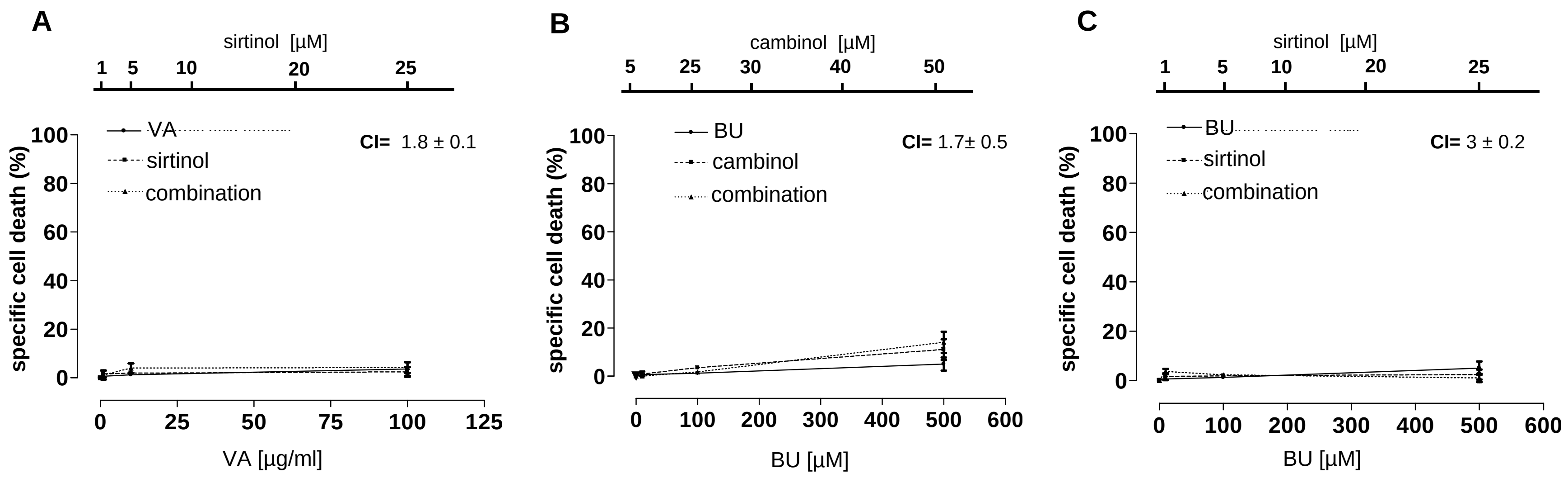

Figure S4. Sirtuin inhibitors and HDAC inhibitors show poor activity and fail to cooperate in healthy PBMCs. A-C, Healthy PMBCs were incubated in 96-well plates with or without sirtinol, cambinol, BU, or VA at the indicated concentrations. Viability was assessed 48 h later by PI cell staining and flow cytometry. CI values refer to the highest drug concentrations used. Results are means ± SD of three independent experiments with three different donors.
