# Supplementary material for: Synergistic Interactions between HDAC and Sirtuin Inhibitors in Human Leukemia Cells
Source: PLoS One. 2011 Jul 27;6(7):e22739. doi: 10.1371/journal.pone.0022739 (PMC3144930; doi:10.1371/journal.pone.0022739)
Supplement: Figure S5 — SIRT1 silencing enhances HDAC inhibitor activity in Jurkat cells. A, B. Jurkat cells were transfected with a non-targeting siRNA (cntr siRNA) or with an anti-SIRT1-siRNA. Thereafter, two days later, cells were used for protein lysate preparation or plated in 96-well plates for viability assays. A, SIRT1 and γ-tubulin levels were determined by immunoblotting. B, Cells were incubated in the presence or absence of the indicated concentrations of VA or of BU. Two days later, dead cells were quantified by PI staining and flow cytometry. One representative experiment out of three is presented. *: p<0.05. (PDF) [file pone.0022739.s005.pdf]

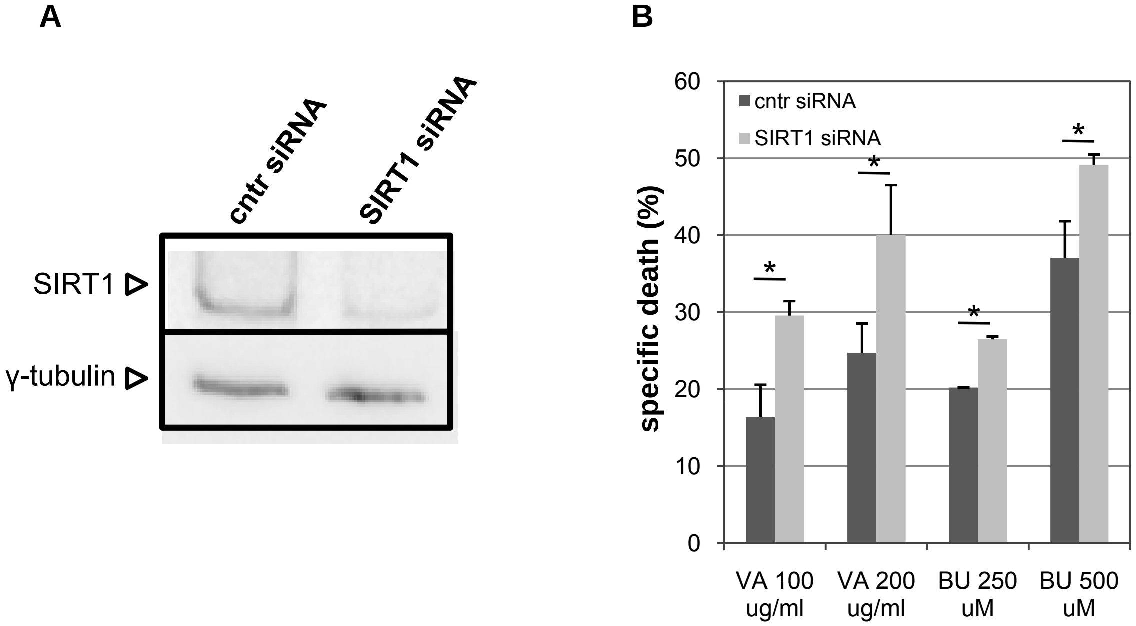

Figure S5. SIRT1 silencing enhances HDAC inhibitor activity in Jurkat cells. A, B. Jurkat cells were transfected with non-targeting siRNAs (cntr siRNA) or anti-SIRT1-siRNAs. Thereafter, two days later, cells were used for protein lysate preparation or plated in 96-well plates for viability assays. A, SIRT1 and γ-tubulin levels were determined by immunoblotting. B, Cells were incubated in the presence or absence of the indicated concentrations of VA or of BU. Two days later, dead cells were quantified by PI staining and flow cytometry. One representative experiment out of three is presented. *: $p<0.05$.
