# Supplementary material for: Synergistic Interactions between HDAC and Sirtuin Inhibitors in Human Leukemia Cells
Source: PLoS One. 2011 Jul 27;6(7):e22739. doi: 10.1371/journal.pone.0022739 (PMC3144930; doi:10.1371/journal.pone.0022739)
Supplement: Figure S6 — SIRT1 expression in primary leukemia cells and in leukemia cell lines. A, RNA was extracted from freshly isolated normal PBMCs (n = 10), primary B-CLL cells (n = 36), AML cells (n = 11), and from the cell lines U937, Jurkat, and 697. SIRT1 levels were determined by Q-PCR. SIRT1 levels in primary leukemia samples (A) and in cell lines (B) were compared to the mean value obtained in PBMCs using the 2−ΔΔCT method. (PDF) [file pone.0022739.s006.pdf]

Figure S6, Cea et al.

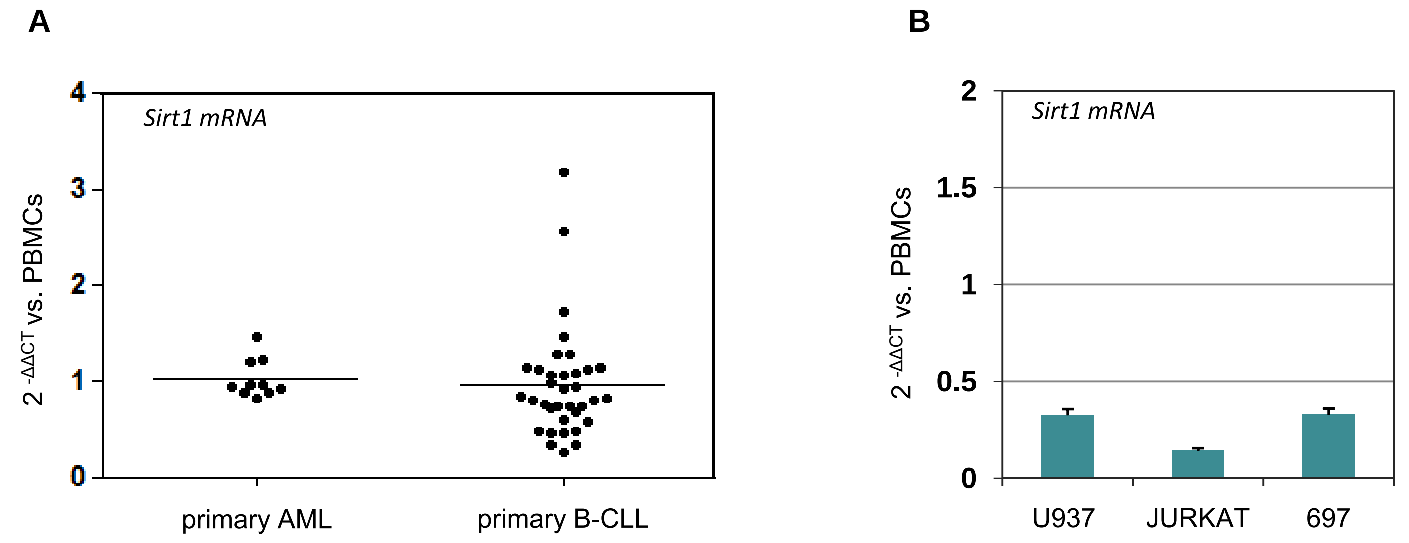

Figure S6. SIRT1 expression in primary leukemia cells and in leukemia cell lines. A, RNA was extracted from freshly isolated normal PBMCs (n = 10), primary B-CLL cells (n = 36), AML cells (n = 11), and from the cell lines U937, Jurkat, and 697. SIRT1 levels were determined by Q-PCR. SIRT1 levels in primary leukemia samples (A) and in cell lines (B) were compared to the mean value obtained in PBMCs using the $2^{-\Delta\Delta CT}$ method.
