# Supplementary material for: Synergistic Interactions between HDAC and Sirtuin Inhibitors in Human Leukemia Cells
Source: PLoS One. 2011 Jul 27;6(7):e22739. doi: 10.1371/journal.pone.0022739 (PMC3144930; doi:10.1371/journal.pone.0022739)
Supplement: Figure S7 — Correlation between SIRT1 expression and activity of the combination sirtuin/HDAC inhibitors in leukemia cells. Primary B-CLL cells were plated in 96 well plates and treated with or without 100 µg/ml VA, 500 µM BU, 50 µM cambinol, or their combinations. Dead cells were enumerated two days later by PI staining and flow cytometry. The correlation between the CI (A, B) or the overall cytotoxic activity (C, D) of each drug combination and SIRT1 expression was assessed by Pearson correlation coefficient. (PDF) [file pone.0022739.s007.pdf]

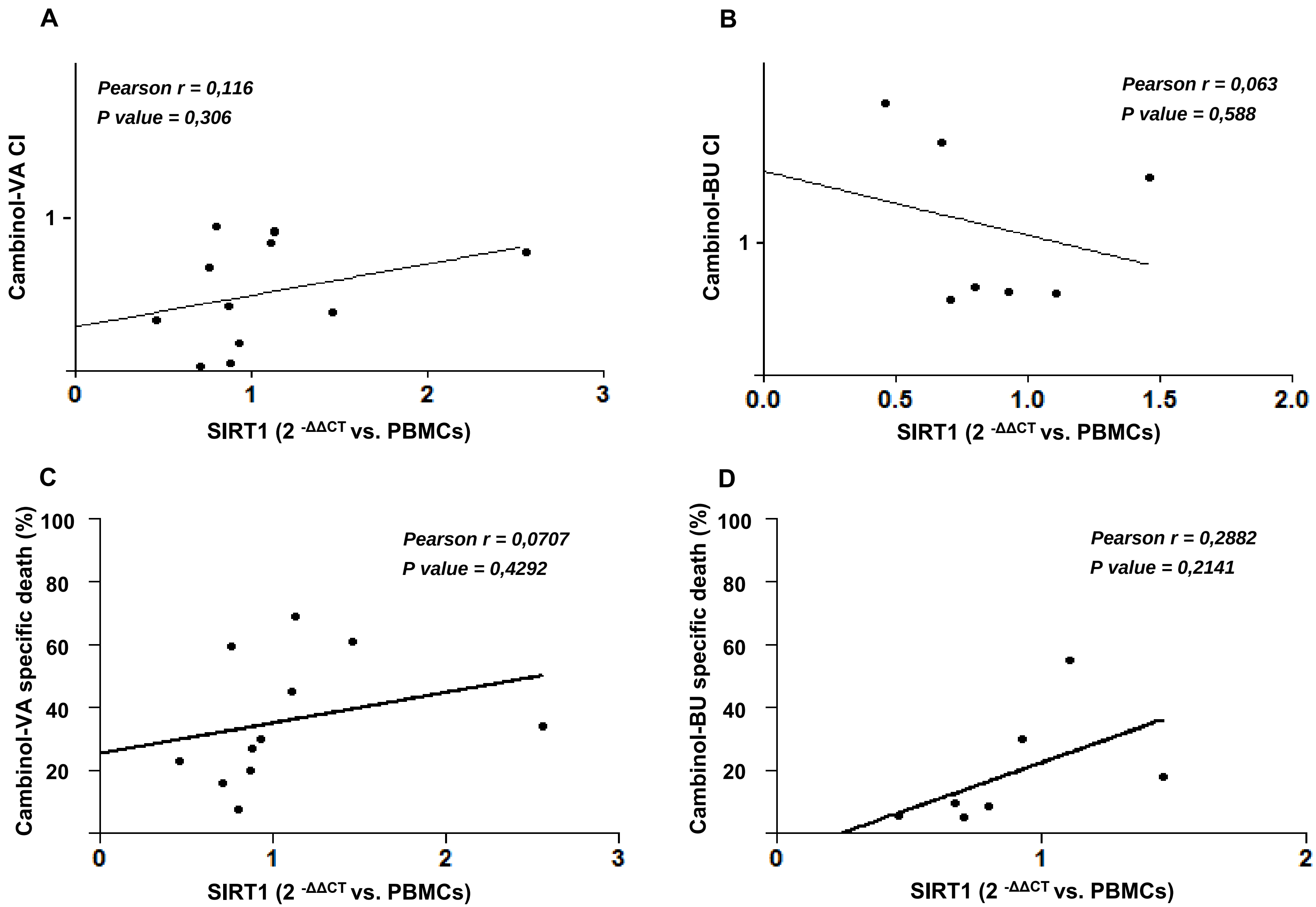


**Figure S7. Correlation between SIRT1 expression and activity of the combination sirtuin/HDAC inhibitors in leukemia cells**. Primary B-CLL cells were plated in 96 well plates and treated with or without 100 µg/ml VA, 500 µM BU, 50 µM cambinol, or their combinations. Dead cells were enumerated two days later by PI staining and flow cytometry. The correlation between the CI (A, B) or the overall cytotoxic activity (C, D) of each drug combination and SIRT1 expression was assessed by Pearson correlation coefficient.
