# Supplementary material for: Synergistic Interactions between HDAC and Sirtuin Inhibitors in Human Leukemia Cells
Source: PLoS One. 2011 Jul 27;6(7):e22739. doi: 10.1371/journal.pone.0022739 (PMC3144930; doi:10.1371/journal.pone.0022739)
Supplement: Figure S8 — Correlation between SIRT1 expression and activity of the combination FK866/HDAC inhibitors in leukemia cells. Primary B-CLL cells were treated with or without 10 nM FK866, 100 µg/ml VA, 500 µM BU, or their combination. Dead cells were enumerated four days later by PI staining and flow cytometry. The correlation between the CI (A, B) or the overall cytotoxic activity (C, D) of each drug combination and SIRT1 expression was assessed by Pearson correlation coefficient. (PDF) [file pone.0022739.s008.pdf]

Figure S8, Cea et al.

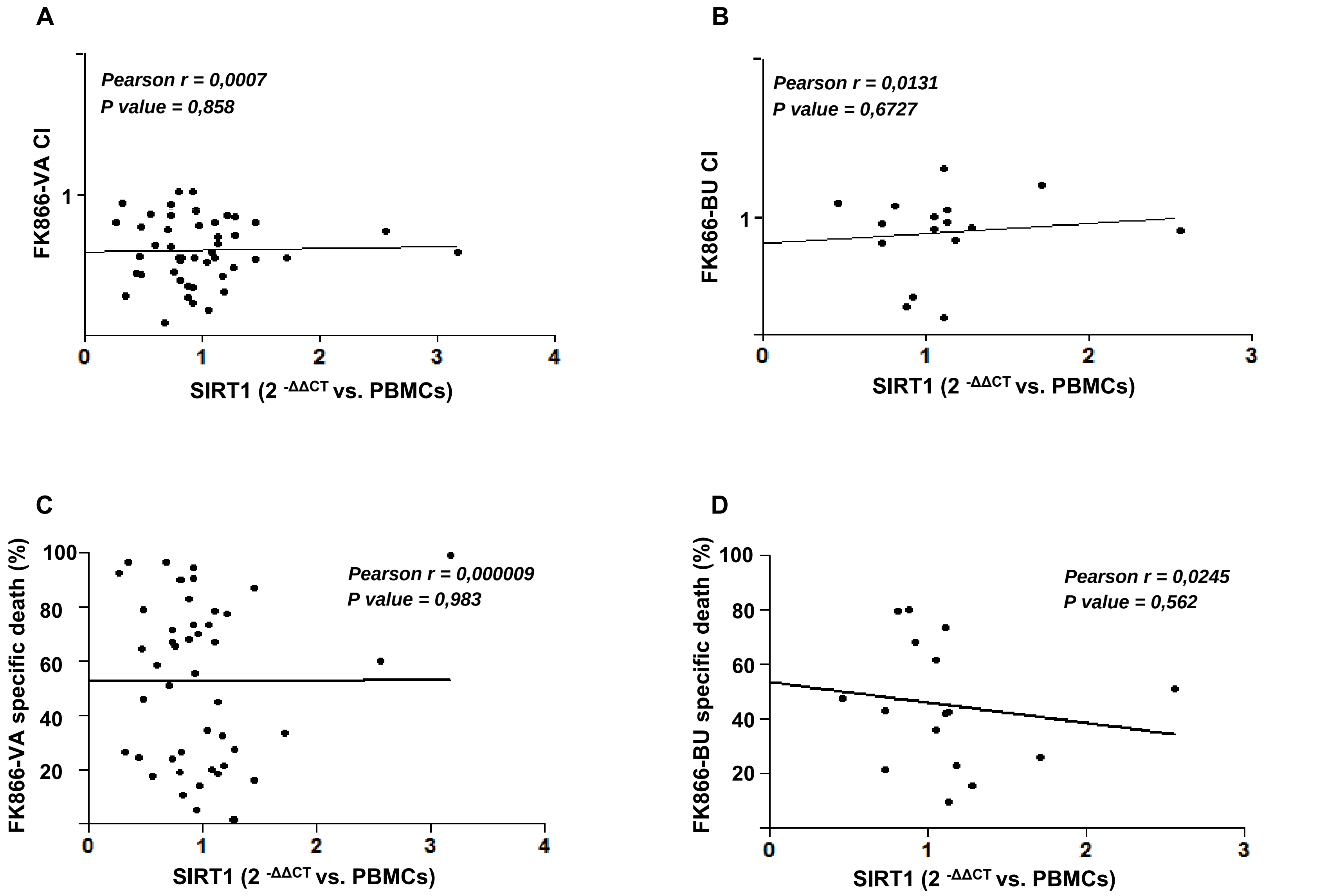

Figure S8. Correlation between SIRT1 expression and activity of the combination FK866/HDAC inhibitors in leukemia cells. Primary B-CLL cells were treated with or without 10 nM FK866, 100 μg/ml VA, 500 μM BU, or their combination. Dead cells were enumerated four days later by PI staining and flow cytometry. The correlation between the CI (A, B) or the overall cytotoxic activity (C, D) of each drug combination and SIRT1 expression was assessed by Pearson correlation coefficient.
