# Supplementary material for: Synergistic Interactions between HDAC and Sirtuin Inhibitors in Human Leukemia Cells
Source: PLoS One. 2011 Jul 27;6(7):e22739. doi: 10.1371/journal.pone.0022739 (PMC3144930; doi:10.1371/journal.pone.0022739)
Supplement: Figure S9 — zVAD-fmk reduces cell death in response to sirtuin and HDAC inhibitors in leukemia cells. A, Jurkat cells were pre-incubated for 1 h with or without 100 µM zVAD-fmk. Thereafter, 100 µg/ml VA, 30 µM sirtinol, 75 µM EX527, or their combinations were added as indicated. Viability was assessed two days later by PI staining and flow cytometry. B–D, 697 cells were pre-incubated for 1 h with or without 100 µM zVAD-fmk. Thereafter, 100 µg/ml VA, 30 µM sirtinol, or their combination were added as indicated. Two days later, cells were imaged by light microscopy (D), dead cells were enumerated by PI staining and flow cytometry (B), and hypodiploid cell nuclei were counted by PI staining of isolated cell nuclei and flow cytometry (C). (PDF) [file pone.0022739.s009.pdf]

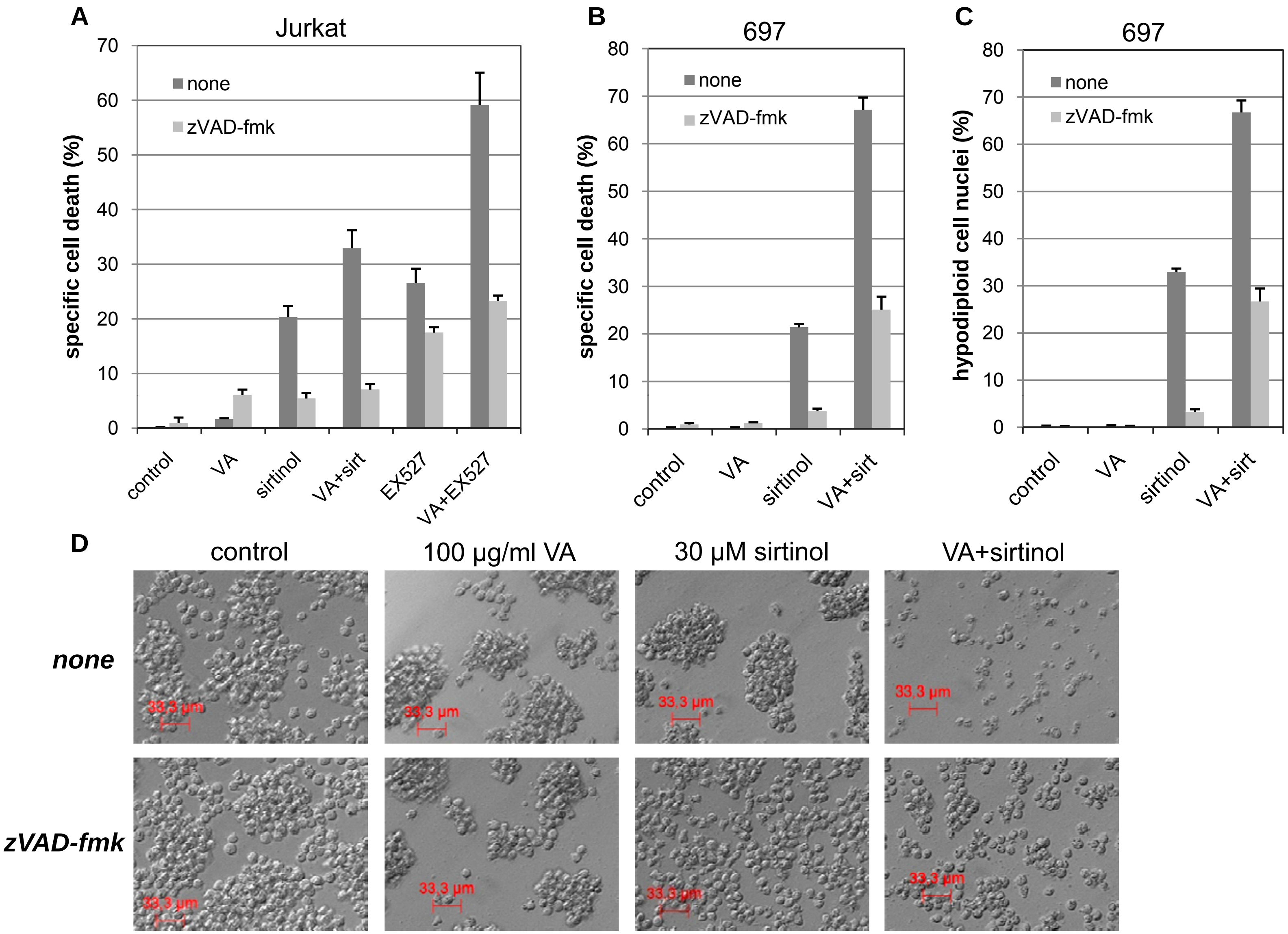

Figure S9. zVAD-fmk reduces cell death in response to sirtuin and HDAC inhibitors in leukemia cells. A, Jurkat cells were pre-incubated for 1 h with or without 100 µM zVAD-fmk. Thereafter, 100 µg/ml VA, 30 µM sirtinol, 75 µM EX527, or their combinations were added as indicated. Viability was assessed two days later by PI staining and flow cytometry. B-D, 697 cells were pre-incubated for 1 h with or without 100 µM zVAD-fmk. Thereafter, 100 µg/ml VA, 30 µM sirtinol, or their combination were added as indicated. Two days later, cells were imaged by light microscopy (D), dead cells were enumerated by PI staining and flow cytometry (B), and hypodiploid cell nuclei were counted by PI staining of isolated cell nuclei and flow cytometry (C).
