# Supplementary material for: Synergistic Interactions between HDAC and Sirtuin Inhibitors in Human Leukemia Cells
Source: PLoS One. 2011 Jul 27;6(7):e22739. doi: 10.1371/journal.pone.0022739 (PMC3144930; doi:10.1371/journal.pone.0022739)
Supplement: Figure S10 — VA induces Bax upregulation in leukemia cell lines, but not in healthy PBMCs. A, 3×10^6^ Jurkat, U937, and 697 cells were plated in 6-well plates in the presence or absence of 100 µg/ml VA. Two days later, cells were harvested, washed and used for protein lysate preparation. Bax and γ-tubulin levels were determined by immunoblotting. B, 107 PBMCs were plates in 3 ml medium in 6-well plates in the presence or absence of the indicated concentrations of VA. Two days later, cells were harvested, washed, and used for cell lysate preparation. Bax and γ-tubulin expression were determined by immunoblotting. A, B one representative experiment out of three is shown. (PDF) [file pone.0022739.s010.pdf]

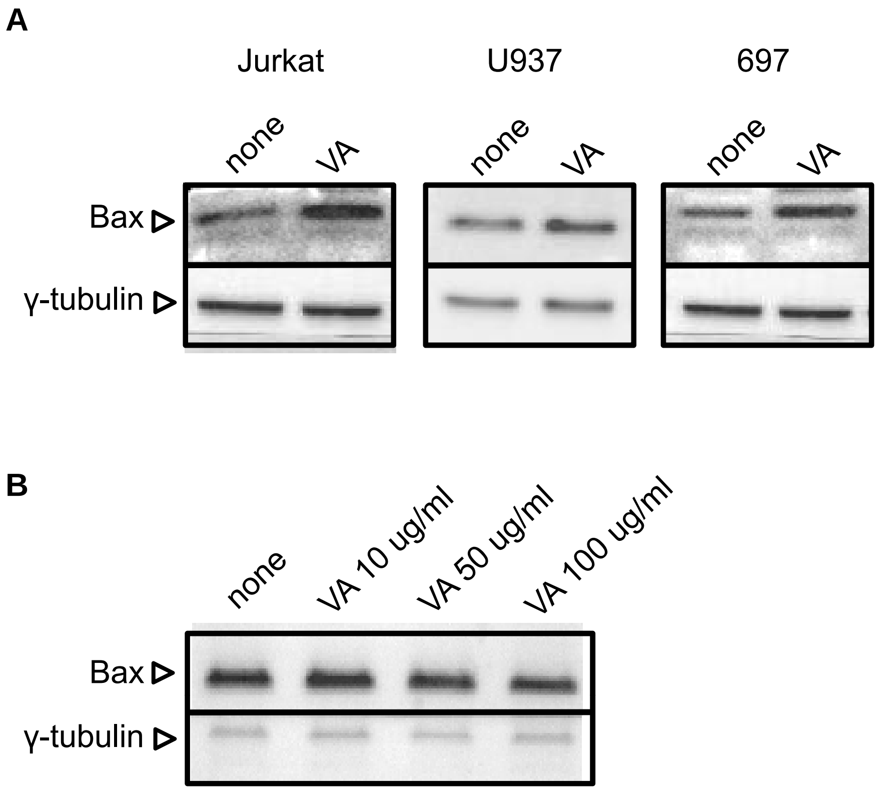

Figure S10. VA induces Bax upregulation in leukemia cell lines but not in healthy PBMCs. A, 3 x 10^6 Jurkat, U937, and 697 cells were plated in 6-well plates in the presence or absence of 100 µg/ml VA. Two days later, cells were harvested, washed and used for protein lysate preparation. Bax and γ-tubulin levels were determined by immunoblotting. B, 10^6 PBMCs were plates in 3 ml medium in 6-well plates in the presence or absence of the indicated concentrations of VA. Two days later, cells were harvested, washed, and used for cell lysate preparation. Bax and γ-tubulin expression were determined by immunoblotting. A, B one representative experiment out of three is shown.
