# Supplementary material for: Synergistic Interactions between HDAC and Sirtuin Inhibitors in Human Leukemia Cells
Source: PLoS One. 2011 Jul 27;6(7):e22739. doi: 10.1371/journal.pone.0022739 (PMC3144930; doi:10.1371/journal.pone.0022739)
Supplement: Figure S11 — FK866-mediated NAD+ depletion mediates FK866's cytotoxic activity. A, Primary B-CLL and AML cells were incubated in 24-well plates in the presence or absence of 10 nM FK866, 100 µg/ml VA, 500 µM BU, or their combination. 48 h later, NAD+ levels were determined by enzymatic cycling assay. NAD+ values were normalized to protein content (expressed in mg). B, Primary AML cells were plated in 96-well plates and incubated with 100 nM FK866. NAD+ was added to the medium every 12 h in order to achieve the indicated concentrations. Viability was quantified after 96 h of incubation by PI staining and flow-cytometry. (PDF) [file pone.0022739.s011.pdf]

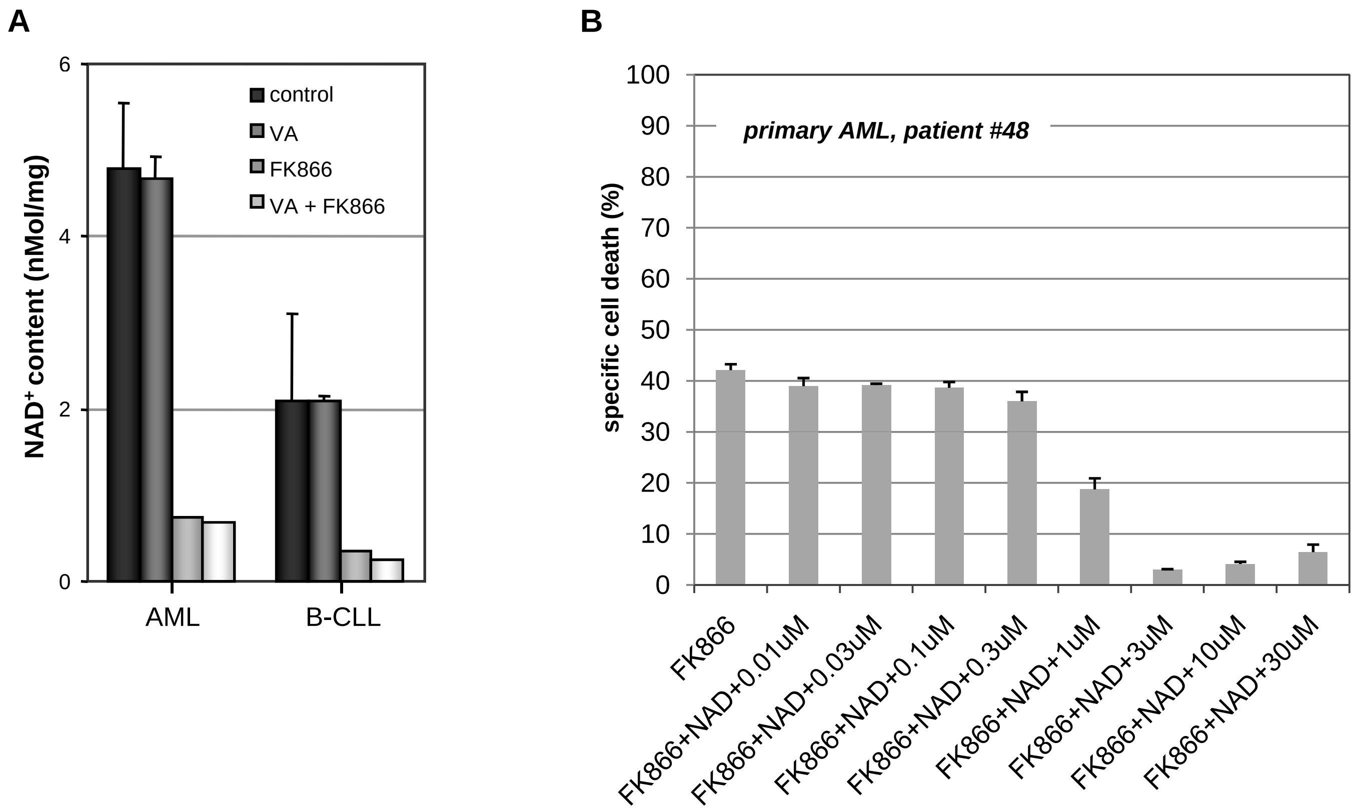

Figure S11. FK866-mediated NAD$^+$ depletion mediates FK866's cytotoxic activity. A, Primary B-CLL and AML cells were incubated in 24-well plates in the presence or absence of 10 nM FK866, 100 μg/ml VA, 500 μM BU, or their combination. 48 h later, NAD$^+$ levels were determined by enzymatic cycling assay. NAD$^+$ values were normalized to protein concentrations (expressed in mg). B, Primary AML cells were plated in 96-well plates and incubated with 100 nM FK866. NAD$^+$ was added to the medium every 12 h in order to achieve the indicated concentrations. Viability was quantified after 96 h of incubation by PI staining and flow-cytometry.
