# Supplementary material for: Synergistic Interactions between HDAC and Sirtuin Inhibitors in Human Leukemia Cells
Source: PLoS One. 2011 Jul 27;6(7):e22739. doi: 10.1371/journal.pone.0022739 (PMC3144930; doi:10.1371/journal.pone.0022739)
Supplement: Figure S12 — FK866 and HDAC inhibitors synergistically kill primary B-CLL cells. Primary B-CLL cells were incubated with or without FK866 at the indicated concentrations for 48 h. Thereafter, VA or vorinostat were added at the indicated concentrations. Viability was assessed 48 h later by PI cell staining and flow cytometry. CI values refer to the highest drug concentrations used. CICTs for each drug combination are presented in the lower insets. (PDF) [file pone.0022739.s012.pdf]

Primary B-CLL cells, patient #36

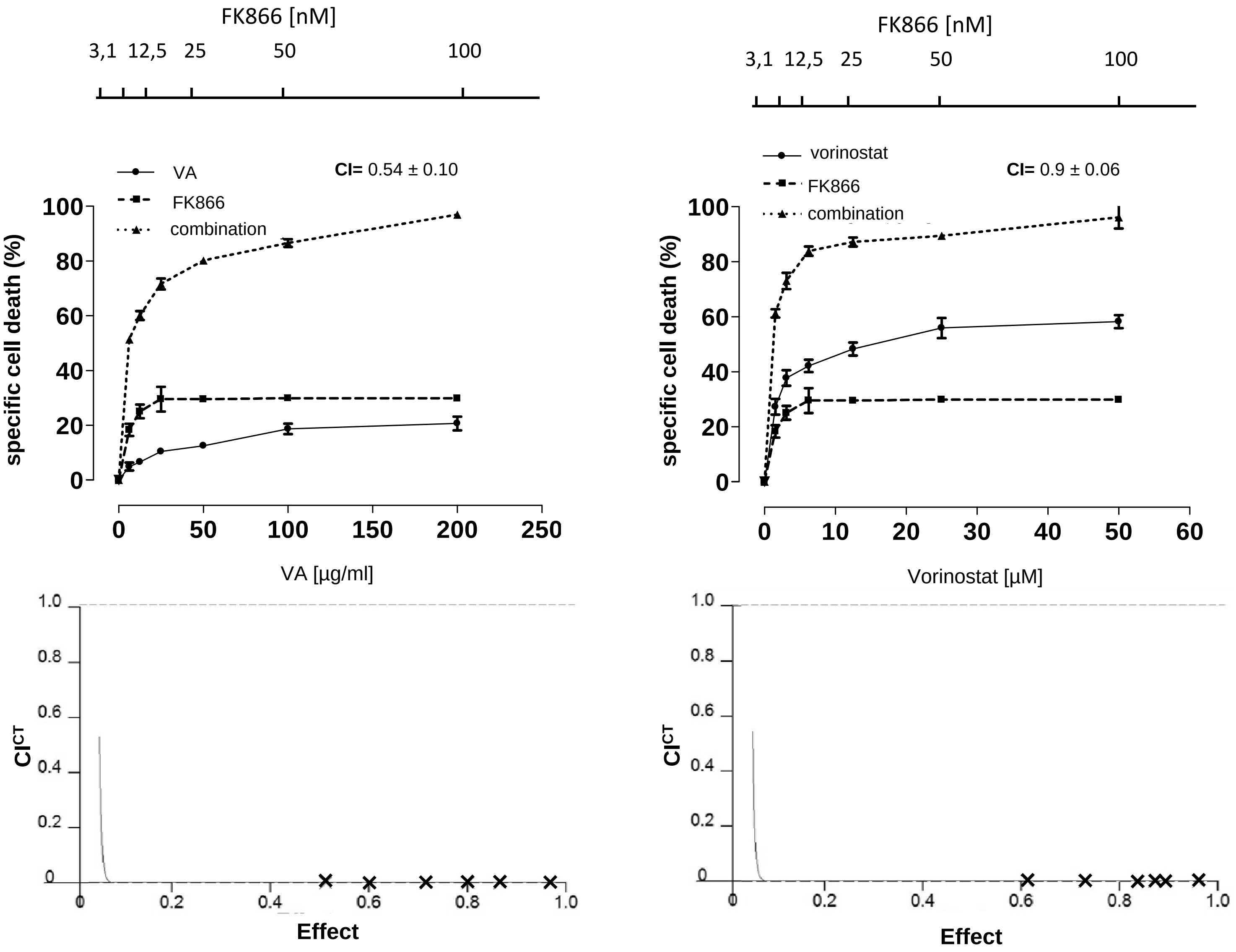

Figure S12. FK866 and HDAC inhibitors synergistically kill primary B-CLL cells. Primary B-CLL cells were incubated with or without FK866 at the indicated concentrations for 48 h. Thereafter, VA or vorinostat were added at the indicated concentrations. Viability was assessed 48 h later by PI cell staining and flow cytometry. CI values refer to the highest drug concentrations used. CI^{CT}s for each drug combination are presented in the lower insets.
