# Supplementary material for: Synergistic Interactions between HDAC and Sirtuin Inhibitors in Human Leukemia Cells
Source: PLoS One. 2011 Jul 27;6(7):e22739. doi: 10.1371/journal.pone.0022739 (PMC3144930; doi:10.1371/journal.pone.0022739)
Supplement: Figure S13 — Synergistic interaction between FK866 and HDAC inhibitors in the AML cell line U937. U937 cells were incubated with or without FK866 at the indicated concentrations for 48 h. Thereafter, VA, vorinostat, or BU were added at the indicated concentrations. Viability was assessed 48 h later by PI cell staining and flow cytometry. CI values refer to the highest drug concentrations used. CICTs for each drug combination are shown in the lower insets. (PDF) [file pone.0022739.s013.pdf]

U937 cells

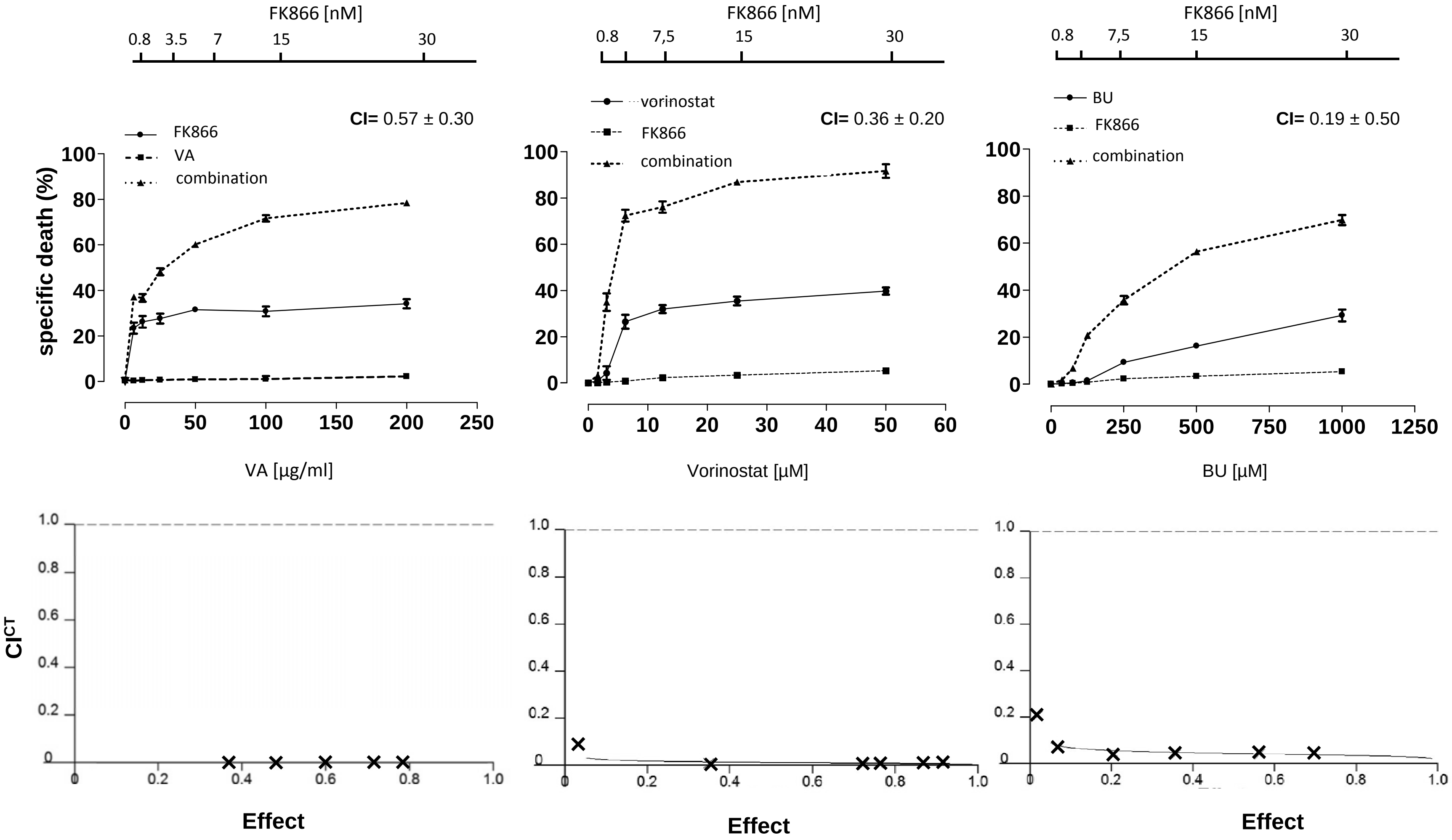

Figure S13. Synergistic interaction between FK866 and HDAC inhibitors in the AML cell line U937. U937 cells were incubated with or without FK866 at the indicated concentrations for 48 h. Thereafter, VA, vorinostat, or BU were added at the indicated concentrations. Viability was assessed 48 h later by PI cell staining and flow cytometry. CI values refer to the highest drug concentrations used. CI^{CT}s for each drug combination are shown in the lower insets.
