# Supplementary material for: Synergistic Interactions between HDAC and Sirtuin Inhibitors in Human Leukemia Cells
Source: PLoS One. 2011 Jul 27;6(7):e22739. doi: 10.1371/journal.pone.0022739 (PMC3144930; doi:10.1371/journal.pone.0022739)
Supplement: Figure S14 — Synergistic interaction between FK866 and HDAC inhibitors in Jurkat cells. Jurkat cells were incubated with or without FK866 at the indicated concentrations for 48 h. Thereafter, VA, vorinostat, or BU were added at the indicated concentrations. Viability was assessed 48 h later by PI cell staining and flow cytometry. CI values refer to the highest drug concentrations used. CICTs for each drug combination are presented in the lower insets. (PDF) [file pone.0022739.s014.pdf]

Figure S14, Cea et al.

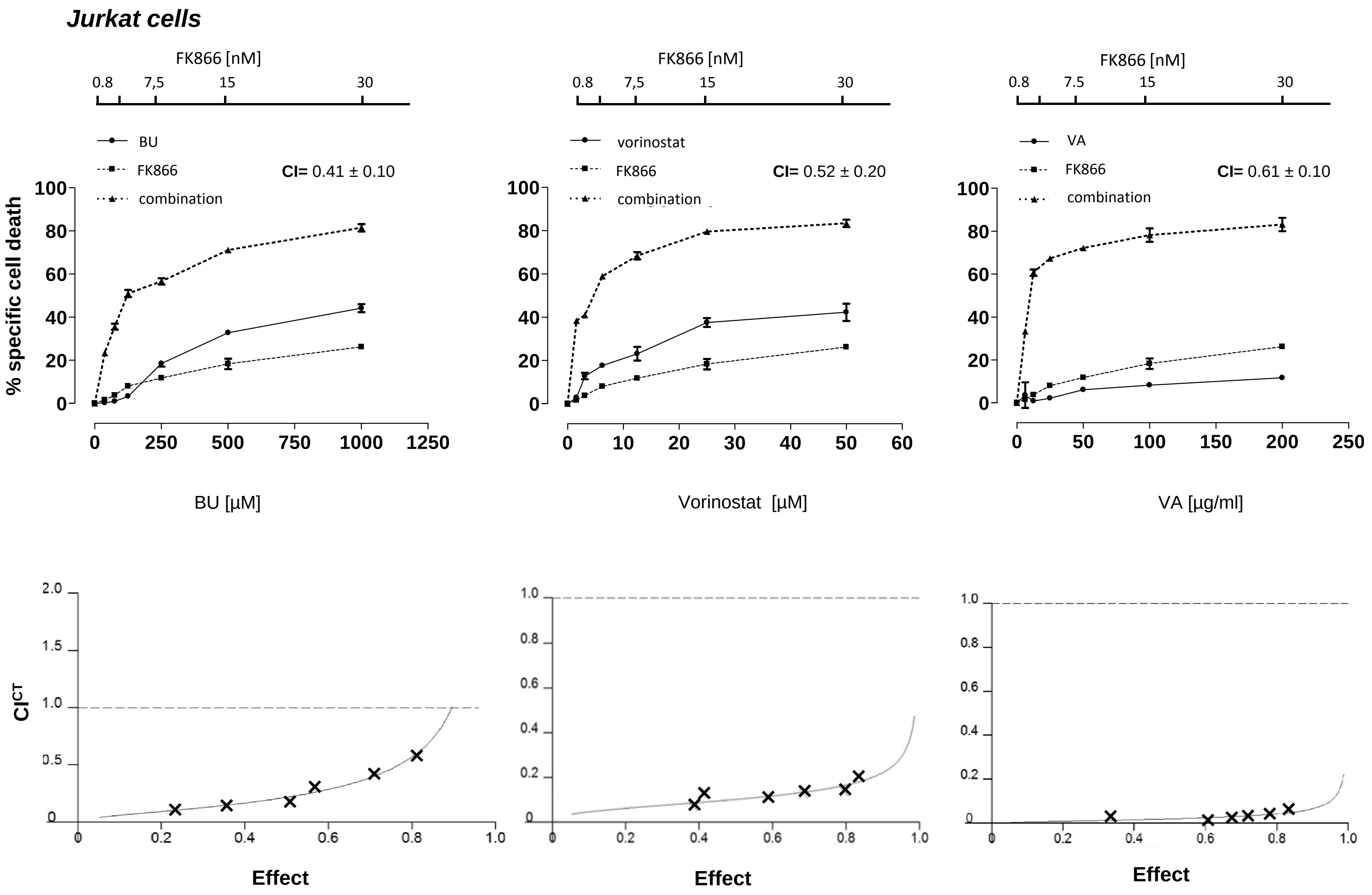

Figure S14. Synergistic interaction between FK866 and HDAC inhibitors in Jurkat cells. Jurkat cells were incubated with or without FK866 at the indicated concentrations for 48 h. Thereafter, VA, vorinostat, or BU were added at the indicated concentrations. Viability was assessed 48 h later by PI cell staining and flow cytometry. CI values refer to the highest drug concentrations used. CI^{CT}s for each drug combination are presented in the lower insets.
