# Supplementary material for: Synergistic Interactions between HDAC and Sirtuin Inhibitors in Human Leukemia Cells
Source: PLoS One. 2011 Jul 27;6(7):e22739. doi: 10.1371/journal.pone.0022739 (PMC3144930; doi:10.1371/journal.pone.0022739)
Supplement: Figure S15 — Synergistic interaction between FK866 and VA in 697 pre-B-cell leukemia cells. 697 cells were incubated with or without FK866 at the indicated concentrations for 48 h. Thereafter, VA was added at the indicated concentrations. Viability was assessed 48 h later by PI cell staining and flow cytometry. CI values refer to the highest drug concentrations used. CICTs are shown in the lower inset. (PDF) [file pone.0022739.s015.pdf]

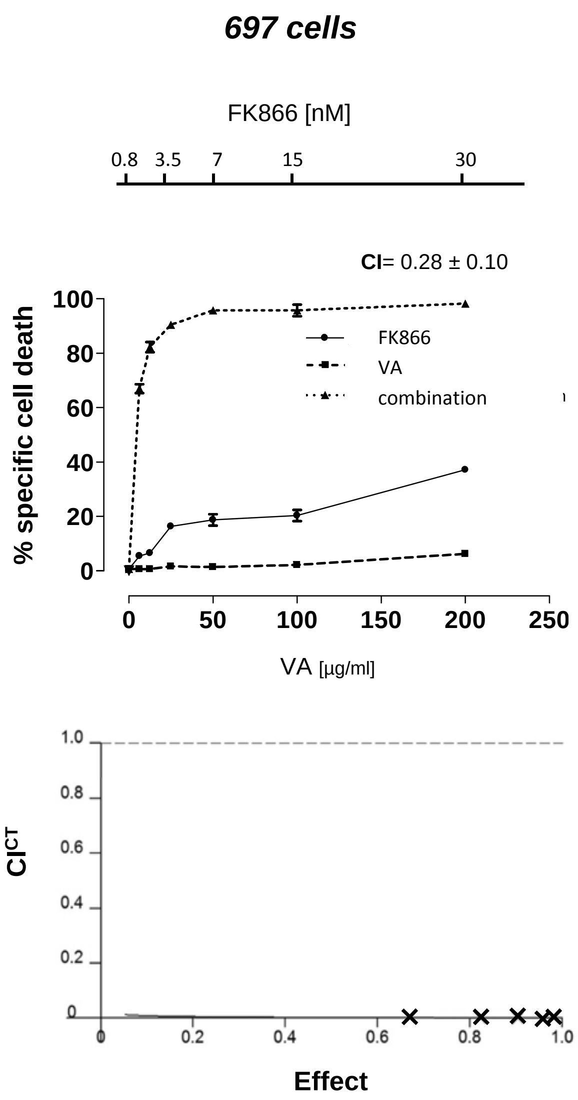


**Figure S15. Synergistic interaction between FK866 and VA in 697 pre-B-cell leukemia cells.** 697 cells were incubated with or without FK866 at the indicated concentrations for 48 h. Thereafter, VA was added at the indicated concentrations. Viability was assessed 48 h later by PI cell staining and flow cytometry. CI values refer to the highest drug concentrations used. $CI^{CT}$s are shown in the lower inset.
