# Supplementary material for: Synergistic Interactions between HDAC and Sirtuin Inhibitors in Human Leukemia Cells
Source: PLoS One. 2011 Jul 27;6(7):e22739. doi: 10.1371/journal.pone.0022739 (PMC3144930; doi:10.1371/journal.pone.0022739)
Supplement: Table S1 — Synergistic interactions between cambinol and VA in primary leukemia cells. Primary B-CLL (#3, #9, #11, #12, #13, #19, #24, #27, #33) or AML (#41, #42, #46) cells were plated in 96 well plates and stimulated with 100 µg/ml VA, cambinol (camb.) at the indicated concentrations, or their combinations. Specific cell death was detected four days later by flow cytometry. CIs are indicated in parenthesis. ND: not determined. (PDF) [file pone.0022739.s016.pdf]

Table S1. Synergistic interactions between cambinol and VA in primary leukemia cells

Patient no.	VA	50 µM camb.	25 µM camb.	5 µM camb.	VA+ 50 µM camb.	VA+ 25 µM camb.	VA+ 5 µM camb.
#3	16,02	10,43	9,56	8,45	34 (0,77)	26,78 (0,95)	25,06 (0,97)
#9	2,51	43,95	27,58	5,84	59,61 (0,77)	44,47 (0,67)	27,09 (0,30)
#11	0,11	0,46	0,22	0,14	16,06 (0,03)	13,86 (0,01)	2,65 (0,22)
#12	0,77	6,91	1,08	0,45	23,1 (0,33)	16,45 (0,06)	11,42 (0,64)
#13	19,93	17,72	11,23	6,23	45,25 (0,83)	40,01 (0,77)	32,56 (0,8)
#19	0,8	22,84	3,87	1,54	61,28 (0,38)	11,65 (0,33)	4,61 (5,28)
#24	27,85	34,76	23	18,76	68,97 (0,90)	ND	48,65 (0,95)
#27	0,79	4,83	3,65	2,65	29,87 (0,18)	22,65 (0,15)	10,06 (0,74)
#33	5,41	1,42	0,64	0,4	7,35 (0,93)	6,19 (0,97)	5,16 (1,12)
#41	6	2,34	1,35	1,54	19,87 (0,42)	16,79 (0,08)	15 (0,25)
#42	1,14	0,42	5,66	4,56	26,87 (0,05)	24,08 (0,23)	9,76 (0,51)
#46	5	18,3	4,3	1,85	49,5 (0,47)	42 (0,22)	14,2 (0,65)

Primary B-CLL (#3, #9, #11, #12, #13, #19, #24, #27, #33) or AML (#41, #42, #46) cells were plated in 96 well plates and stimulated with 100 µg/ml VA, cambinol (camb.) at the indicated concentrations, or their combinations. Specific cell death was detected four days later by flow cytometry. CIs are indicated in parenthesis. ND: not determined.
