# Supplementary material for: Synergistic Interactions between HDAC and Sirtuin Inhibitors in Human Leukemia Cells
Source: PLoS One. 2011 Jul 27;6(7):e22739. doi: 10.1371/journal.pone.0022739 (PMC3144930; doi:10.1371/journal.pone.0022739)
Supplement: Table S2 — Synergistic interactions between cambinol and BU in primary leukemia cells. Primary B-CLL cells were plated in 96 well plates and stimulated with 500 µM BU, cambinol (camb.) at the indicated concentrations, or their combinations. Specific cell death was detected four days later by flow cytometry. CIs are indicated in parenthesis. (PDF) [file pone.0022739.s017.pdf]

Table S2. Synergistic interactions between cambinol and BU in primary leukemia cells

Patient no.	BU	50 µM camb.	25 µM camb.	5 µM camb.	BU+ 50 µM camb.	BU+ 25 µM camb.	BU+ 5 µM camb.
#9	11,28	2,95	1,58	0,84	19,61 (0,72)	14,47 (0,88)	12,09 (1)
#11	2,07	0,46	0,22	0,14	5,26 (0,48)	3,23 (0,70)	2,53 (0,87)
#12	2,6	6,91	1,084	0,4	15,48 (0,61)	6,11 (0,60)	3,43 (0,87)
#13	1,23	27,72	0,11	0,16	54,86 (0,52)	23,54 (0,05)	2,01 (0,69)
#19	0,38	22,84	3,87	1,54	28,33 (0,81)	8,13 (0,52)	3,55 (0,54)
#27	3,41	12,89	9,87	2,34	30,3 (0,53)	17,31 (0,76)	7,79 (0,73)
#33	3,4	1,42	0,64	0,4	8,49 (0,56)	7,92 (0,51)	4,03 (0,94)

Primary B-CLL cells were plated in 96 well plates and stimulated with 500 µM BU, cambinol (camb.) at the indicated concentrations, or their combinations. Specific cell death was detected four days later by flow cytometry. CIs are indicated in parenthesis.
