# Supplementary material for: Synergistic Interactions between HDAC and Sirtuin Inhibitors in Human Leukemia Cells
Source: PLoS One. 2011 Jul 27;6(7):e22739. doi: 10.1371/journal.pone.0022739 (PMC3144930; doi:10.1371/journal.pone.0022739)
Supplement: Table S3 — Synergistic interactions between sirtinol and VA in primary leukemia cells. Primary B-CLL (#3, #9, #11, #12, #13, #19, #24, #27, #33, #36) or AML (#41, #42, #46) cells were plated in 96 well plates and stimulated with 100 µg/ml VA, sirtinol (sirt.) at the indicated concentrations, or their combinations. Specific cell death was detected four days later by flow cytometry. CIs are indicated in parenthesis. (PDF) [file pone.0022739.s018.pdf]

Table S3. Synergistic interactions between sirtinol and VA in primary leukemia cells

Patient nr.	VA	25 µM sirt.	5 µM sirt.	1 µM sirt.	VA+ 25 µM sirt.	VA+ 5 µM sirt.	VA+ 1 µM sirt.
#3	16,02	51,08	40,8	29,06	87,6 (0,76)	64,96 (0,87)	55,65 (0,81)
#9	2,51	16,55	1,84	0,369	45,16 (0,56)	33,79 (0,24)	16,51 (0,41)
#11	0,11	4,31	0,64	0,16	14,29 (0,30)	8,1 (0,09)	3,06 (0,08)
#12	0,77	6,5	1,08	0,4	14,24 (0,51)	9,11 (0,20)	2,43 (0,48)
#13	19,93	3,56	1,76	0,43	34,87 (0,67)	24,81 (0,87)	20,56 (1,14)
#19	0,8	4,25	0,52	0,03	10,55 (0,47)	8,97 (0,14)	6,73 (0,12)
#24	27,85	70,3	2,32	1,76	96,6 (1,01)	56,48 (0,53)	24,86 (1,19)
#27	0,79	3	1,48	1	18,31 (0,21)	15,52 (0,14)	14,99 (0,11)
#33	5,41	21,21	6,06	2,62	38,13 (0,69)	26,49 (0,43)	15,62 (0,51)
#36	11,3	13,5	1,56	0,34	33,56 (0,73)	15 (0,85)	2,54 (4,5)
#41	6	9,52	5,23	4,98	81,6 (0,19)	71,74 (0,15)	66,11 (0,16)
#42	1,14	0,93	0,24	0,21	13,12 (0,15)	10,6 (0,13)	6,3 (0,21)
#46	7,8	6,4	0,54	0,21	57 (0,24)	17,9 (0,46)	1,34 (5,9)

Primary B-CLL (#3, #9, #11, #12, #13, #19, #24, #27, #33, #36) or AML (#41, #42, #46) cells were plated in 96 well plates and stimulated with 100 µg/ml VA, sirtinol (sirt.) at the indicated concentrations, or their combinations. Specific cell death was detected four days later by flow cytometry. CIs are indicated in parenthesis.
