# Supplementary material for: Synergistic Interactions between HDAC and Sirtuin Inhibitors in Human Leukemia Cells
Source: PLoS One. 2011 Jul 27;6(7):e22739. doi: 10.1371/journal.pone.0022739 (PMC3144930; doi:10.1371/journal.pone.0022739)
Supplement: Table S4 — Synergistic interactions between sirtinol and BU in primary leukemia cells. Primary B-CLL (#3, #9, #11, #12, #13, #19, #24, #27, #33) or AML (#41, #42) cells were plated in 96 well plates and stimulated with 500 µM BU, sirtinol (sirt.) at the indicated concentrations, or their combinations. Specific cell death was detected four days later by flow cytometry. CIs are indicated in parenthesis. (PDF) [file pone.0022739.s019.pdf]

Table S4. Synergistic interactions between sirtinol and BU in primary leukemia cells

Patient nr.	BU	25 µM sirt.	5 µM sirt.	1 µM sirt.	BU+ 25 µM sirt.	BU+ 5 µM sirt.	BU+ 1 µM sirt.
#3	6,02	51,08	40,8	29,06	64,89 (0,87)	51,23 (0,91)	42,97 (0,81)
#9	11,28	36,55	1,84	0,369	68,09 (0,70)	51,96 (0,25)	21,55 (0,54)
#11	2,07	4,31	0,64	0,16	13,48 (0,47)	8 (0,33)	2,17 (1,02)
#12	2,6	6,5	1,08	0,4	12,19 (0,74)	8,18 (0,44)	1,42 (2,11)
#13	1,23	3,56	1,76	0,43	19,56 (0,24)	14,76 (0,20)	9,71 (0,17)
#19	0,38	4,25	0,52	0,03	10,38 (0,44)	5,73 (0,15)	2,45 (0,16)
#24	1,34	75,3	2,32	1,76	83,91 (0,91)	31,97 (0,11)	10,43 (0,29)
#27	3,41	3	1,48	1	20,3 (0,31)	10,31 (0,47)	5,91 (0,74)
#33	3,4	21,21	6,06	2,62	36,92 (0,66)	13,03 (0,72)	8,94 (0,67)
#41	2,23	9,52	5,23	4,98	24,78 (0,47)	11,35 (0,65)	9,56 (0,75)
#42	4,78	0,93	0,24	0,21	14,83 (0,38)	10,35 (0,48)	5,62 (0,88)

Primary B-CLL (#3, #9, #11, #12, #13, #19, #24, #27, #33) or AML (#41, #42) cells were plated in 96 well plates and stimulated with 500 µM BU, sirtinol (sirt.) at the indicated concentrations, or their combinations. Specific cell death was detected four days later by flow cytometry. CIs are indicated in parenthesis.
