# Supplementary material for: Synergistic Interactions between HDAC and Sirtuin Inhibitors in Human Leukemia Cells
Source: PLoS One. 2011 Jul 27;6(7):e22739. doi: 10.1371/journal.pone.0022739 (PMC3144930; doi:10.1371/journal.pone.0022739)
Supplement: Table S5 — Synergistic interactions between FK866 and HDAC inhibitors in primary leukemia cells. Primary B-CLL (#1–35) or AML (#37–45) cells were plated in 96 well plates and stimulated with 100 µg/ml VA, 500 µM BU, 10 nM FK866 or their combinations. Specific cell death was detected four days later by PI staining and flow cytometry. CIs are indicated in parenthesis. ND: not determined. (PDF) [file pone.0022739.s020.pdf]

Table S5. Synergistic interactions between FK866 and HDAC inhibitors in primary leukemia cells.

Patient nr.	VA	BU	APO 866	VA+FK866	BU+FK866
1	1,29	3,99	43,53	73,47 (0,61)	61,81 (0,76)
#2	9,08	3,17	5,09	78,72 (0,18)	73,41 (0,12)
#3	2,87	8,41	30,23	60,18 (0,55)	50,92 (0,75)
#4	1,56	5,26	23,45	33,79 (0,74)	26,28 (1,08)
#5	0,67	ND	9,1	17,76 (0,55)	ND
#6	20,86	ND	35,76	65,82 (0,86)	ND
#7	8,43	ND	34,15	94,62 (0,45)	ND
#8	2,9	ND	23,87	26,55 (1,01)	ND
#9	21,4	ND	29,76	96,52 (0,53)	ND
#10	2,01	ND	0,34	24,51 (0,5)	ND
#11	3,56	ND	18,85	50,93 (0,44)	ND
#12	7,26	4,22	41,32	64,35 (0,75)	47,6 (0,95)
#13	9,9	22,9	27,72	67,17 (0,56)	41,89 (0,56)
#14	22,9	26,42	4,76	34,57 (0,8)	36,29 (0,75)
#15	19,69	ND	4,21	45,96 (0,52)	ND
#16	1,1	ND	0,03	1,46 (0,77)	ND
#17	6,64	ND	5,11	13,98 (0,84)	ND
#18	0,21	ND	1,13	1,71 (0,78)	ND
#19	7,55	ND	0,19	16,12 (0,48)	ND
#20	43,11	ND	8,98	96,46 (0,54)	ND
#21	1,07	ND	26,68	99,1 (0,28)	ND
#22	9,16	ND	44,6	89,89 (0,59)	ND
#23	10,96	3,88	28,54	71,81 (0,55)	42.9 (0,66)
#24	10,44	10,94	27,85	45,04 (0,85)	42,67 (0,9)

#25	7,95	9,35	7,85	24,3 (0,65)	21,49 (0,8)
#26	8,92	ND	0,87	10,64 (0,92)	ND
#27	8,81	ND	21,79	55,63 (0,55)	ND
#28	4,78	ND	9,86	26,61 (0,55)	ND
#29	24,78	ND	48,67	78,97 (0,93)	ND
#30	18,04	ND	7,14	58,55 (0,43)	ND
#31	9,21	ND	50	92,51 (0,64)	ND
#32	5,89	ND	10,24	20,16 (0,8)	ND
#33	4,78	1,07	6,89	18,52 (0,63)	9,82 (0,81)
#34	9,77	2,85	13,2	32,81 (0,7)	23,31 (0,68)
#35	4,23	4,56	7,4	27,69 (0,42)	15,47 (0,77)
#36	34,85	ND	20,87	77,77 (0,71)	ND
#37	0,45	ND	3,91	5,12 (0,85)	ND
#38	17,34	ND	1,81	21,76 (0,88)	ND
#39	12,03	ND	15,04	87,32 (0,31)	ND
#40	30,28	32,7	41,95	90,28 (0,8)	79,76 (0,93)
#41	17,89	ND	8,72	68,23 (0,39)	ND
#42	10,45	3,45	12,72	83,24 (0,27)	80 (0,2)
#43	18,32	ND	13,45	90,43(0,35)	ND
#44	8,74	2,37	16,51	73,34(0,34)	68,3 (0,27)
#45	7,21	ND	9,42	70,32(0,23)	ND

Primary B-CLL (#1-35) or AML (#37-45) cells were plated in 96 well plates and stimulated with 100 μg/ml VA, 500 μM BU, 10 nM FK866 or their combinations. Specific cell death was detected four days later by PI staining and flow cytometry. CIs are indicated in parenthesis. ND: not determined.
