# Supplementary material for: Synergistic Interactions between HDAC and Sirtuin Inhibitors in Human Leukemia Cells
Source: PLoS One. 2011 Jul 27;6(7):e22739. doi: 10.1371/journal.pone.0022739 (PMC3144930; doi:10.1371/journal.pone.0022739)
Supplement: Table S6 — Clinical and laboratory features of patients with B-CLL. ND: not determined; Nor, normal; +: trisomy; *:chemonaive patient. (PDF) [file pone.0022739.s021.pdf]

**Table S6. Clinical and laboratory features of patients with B-CLL**

| Patient no. | Sex | Age, y | Cytogenetics | RAI stage | $IgV_H$ | Zap70 | CD38 |
|---|---|---|---|---|---|---|---|
| #1 | M | 71 | Nor | RAI 0 | mutated | Neg | Neg |
| #2* | F | 67 | 13q- | RAI IV | mutated | Pos | Pos |
| #3* | F | 68 | Nor | RAI IV | mutated | Neg | Neg |
| #4 | M | 74 | 17p- | RAI 0 | unmutated | Pos | Pos |
| #5 | M | 76 | Nor | RAI II | mutated | Neg | Neg |
| #6 | M | 65 | ND | RAI IV | mutated | Neg | Neg |
| #7 | M | 66 | 17p- | RAI II | mutated | Pos | Pos |
| #8* | M | 58 | Nor | RAI II | mutated | Neg | Neg |
| #9* | M | 79 | 13q-,17p- | RAI 0 | unmutated | Pos | Pos |
| #10 | M | 72 | Nor | RAI IV | unmutated | Pos | Pos |
| #11* | F | 67 | ND | RAI 0 | mutated | Neg | Neg |
| #12 | F | 63 | 17p- | RAI 0 | mutated | Neg | Neg |
| #13 | M | 77 | 12 + | RAI II | mutated | Neg | Neg |
| #14 | M | 75 | ND | RAI III | unmutated | Pos | Neg |
| #15* | M | 73 | 13q-,11q- | RAI IV | unmutated | Neg | Pos |
| #16 | M | 67 | Nor | RAI 0 | mutated | Neg | Neg |
| #17* | M | 65 | 17p- | RAI II | mutated | Neg | Pos |
| #18 | M | 77 | 12+ | RAI IV | mutated | Neg | Neg |
| #19 | M | 56 | 13q- | RAI III | unmutated | Neg | Neg |
| #20 | M | 73 | Nor | RAI 0 | unmutated | Pos | Pos |
| #21 | M | 78 | 13q-,11q- | RAI 0 | mutated | Neg | Neg |
| #22* | F | 67 | Nor | RAI II | mutated | Pos | Neg |
| #23 | M | 66 | Nor | RAI II | mutated | Neg | Pos |
| #24* | M | 64 | Nor | RAI II | mutated | Neg | Neg |
| #25 | M | 76 | 17p- | RAI III | mutated | Neg | Pos |
| #26* | M | 71 | 13q-,17p- | RAI 0 | unmutated | Neg | Neg |
| #27 | F | 70 | ND | RAI III | unmutated | Pos | Pos |
| #28 | M | 72 | ND | RAI IV | mutated | Pos | Neg |
| #29 | M | 67 | ND | RAI IV | mutated | Pos | Pos |
| #30* | F | 62 | 13q- | RAI 0 | unmutated | Neg | Pos |
| #31* | M | 67 | ND | RAI I | unmutated | Neg | Neg |
| #32 | M | 74 | ND | RAI II | mutated | Pos | Pos |
| #33* | M | 62 | 13q- | RAI 0 | unmutated | Neg | Pos |
| #34 | F | 71 | ND | RAI I | mutated | Neg | Pos |
| #35 | F | 74 | 17p- | RAI 0 | unmutated | Neg | Neg |
| #36 | M | 75 | ND | RAI 0 | unmutated | Neg | Neg |

ND: not determined; Nor, normal; +: trisomy; *:chemonaive patient.
