# Supplementary material for: Synergistic Interactions between HDAC and Sirtuin Inhibitors in Human Leukemia Cells
Source: PLoS One. 2011 Jul 27;6(7):e22739. doi: 10.1371/journal.pone.0022739 (PMC3144930; doi:10.1371/journal.pone.0022739)
Supplement: Table S7 — Clinical and laboratory features of patients with AML. PB: peripheral blood. (PDF) [file pone.0022739.s022.pdf]

Table S7. Clinical and laboratory features of patients with AML

Patient nr.	Sex	Age	Sample source	FAB Stage	% Blasts
#37	M	53	PB	M4	95
#38	F	50	PB	M1	78
#39	M	80	PB	secondary	90
#40	M	78	PB	secondary	80
#41	M	79	PB	secondary	70
#42	F	40	PB	M4	95
#43	M	74	PB	secondary	90
#44	M	45	PB	M5	85
#45	M	68	PB	M4	75
#46	F	70	PB	M0/M1	90
#47	M	90	PB	secondary	95
#48	M	70	PB	M5	90

PB: peripheral blood.
